# Early Optimistic Effect in Periodontology and Implant Dentistry Trials

**DOI:** 10.1177/00220345211025242

**Published:** 2021-07-08

**Authors:** M.C. Menne, G. Seitidis, C.M. Faggion, D. Mavridis, N. Pandis

**Affiliations:** 1 Department of Periodontology and Operative Dentistry, Faculty of Dentistry, University Hospital Münster, Münster, Germany; 2 Department of Primary Education, School of Education, University of Ioannina, Ioannina, Greece; 3 Department of Orthodontics and Dentofacial Orthopedics, Dental School/Medical Faculty, University of Bern, Bern, Switzerland

**Keywords:** bias, systematic reviews, meta-analysis, methods, methodological study, evidence-based dentistry

## Abstract

Differences in effect estimates between early primary trials included in a meta-analysis and the pooled estimate of meta-analysis might indicate potential novelty bias. The objective of this study was to assess the presence of novelty bias in a sample of studies published in periodontology and implant dentistry. On August 7, 2020, we searched the PubMed database for meta-analyses of clinical studies published between August 2015 and August 2020. Meta-analyses with at least 4 primary studies were selected for assessment. We fitted logistic regression models using trial characteristics as predictors to assess the association between these characteristics and 1) the odds of the first trial’s estimate to be included in the meta-analysis confidence interval (CI) and 2) the odds of overlap between the first trial’s CI and the meta-analysis prediction interval (PI). Ninety-two meta-analyses provided data for assessment. In absolute values, 70% of the meta-analyses have a pooled estimate smaller than the corresponding estimate of the first trial, although there was overlap of the CI of estimates from the first trial and the meta-analysis in 87% of the cases. This is probably due to the small number of trials in most meta-analyses and the subsequently large uncertainty associated with the pooled effect estimate. As the number of trials in the meta-analysis increased, the odds of the treatment effect estimate of the first trial to be included in the meta-analysis CI decreased by 15% for every additional trial (odds ratio, 0.85; 95% CI, 0.73 to 0.96). Meta-analytic effect estimates appear to be more conservative than those from the first trial in the meta-analysis. Our findings show evidence of novelty bias in periodontology and implant dentistry; therefore, clinicians should be aware of the risk of making decisions based on the information reported in new trials because of the risk of exaggerated estimates in these trials.

## Introduction

Clinical trials are important for clinical practice; therefore, it is essential that the findings of such studies are trustworthy. Different types of bias can interfere with the results of individual studies assessing therapeutic interventions by distorting the direction and size of the effect estimates (Savovic et al. 2018). Common types of bias in clinical trials include selection, performance, detection, attrition, and reporting bias and are associated with improper methodology applied during the design, conduct, analysis, and reporting of the trial (Persaud and Heneghan 2021). Such biases can hamper trial internal validity, render study results invalid, and compromise health care recommendations and patient care.

It is advocated that health care recommendations should be based on the totality of the available high-quality evidence via synthesis commonly found in the form of systematic reviews with meta-analysis. An issue acting against this principle is publication bias (Dickersin 1990), which refers to the publication or not of trial findings based on the size and direction of the observed treatment effects, a persistent problem despite the increased awareness (Fanelli 2012). In the context of academic success and under the “publish or perish” principle, researchers have strong incentives to publish their results quickly, further contributing to publication bias and false discovery rates (Simmons et al. 2011). Suspicion of publication bias can result in questionable meta-analysis estimates, as the gathering of the totality of the evidence is precluded since some studies with smaller treatment effects may not be published, thus biasing meta-analysis estimates away from the null.

There is great enthusiasm in discovery of new treatments that are expected to improve patient care; however, there is evidence in the literature that strong treatment effects in early and especially small trials can be refuted in consequent trials (Simmons et al. 2011). Such bias where initially strong treatment effects are not confirmed in subsequent studies has been coined “novelty bias” and may be the result of a combination of biases observed during the design, conduct, analysis, and reporting of a trial (Ioannidis 2008; Persaud and Heneghan 2021). As it is common for meta-analyses to have few studies, the possibility of early trials with exaggerated results is likely to inflate the pooled effect. Novelty bias, which implies that results of early trials can be exaggerated, can pose a problem in patient care if clinicians incorporate interventions based solely on the results of early trial results. Therefore, clinicians should be cautious into adopting new treatments based on limited data.

Ioannidis (2005) examined such controversies in 49 highly cited studies (>1,000 citations) published in 3 major general clinical journals or high–impact factor specialty journals between 1990 and 2003 covering a range of medical interventions. Among the 49 first trials, 45 concluded that the intervention was effective. The results of 7 of 45 (16%) were contradicted by subsequent studies; in another 7 of 45 (16%), the effects of the first trial were stronger than those of subsequent studies; 20 of 45 (44%) were replicated; and 11 of 45 (24%) remained largely unchallenged. Novelty bias has been also identified for some lipid-lowering antiglaucoma drugs (Gehr et al. 2006), cancer treatment (Salanti et al. 2010), interferon hepatitis C treatments (Tinè et al. 2017), and antidepressants (Cipriani et al. 2018). Using cumulative network meta-analysis, Luo et al. (2021) indicated initially stronger effects for antidepressants, which stabilized, however, and the confidence in the evidence increased with the addition of new studies over time.

A relevant problem is the reproducibility of study findings, as recently highlighted in the field of psychology where reproducibility after replication of initial studies was relatively low (Open Science Collaboration 2015). In 100 studies, replication effects were half the magnitude of first study effects. In 98% of the first studies, the results were statistically significant, whereas 36% of replications had statistically significant results; 47% of first effect sizes were included in the 95% confidence interval (CI) of the replication effect size. In 39% of the replicated studies, the first study effects were subjectively rated to have replicated the original result.

Systematic assessment of novelty bias in the field of dentistry is very limited, with 1 study assessing temporal trends in the efficacy of periodontal regeneration where no temporal trends were identified (Tu et al. 2008). Therefore, the aim of this meta-epidemiologic study, as the evidence accumulates, was to examine whether there is evidence of novelty bias in treatment effects of interventions relevant to periodontology and implant dentistry.

## Materials and Methods

### Eligibility Criteria

We included meta-analyses of clinical studies on different types of therapeutic interventions related to periodontology and implant dentistry. Systematic reviews not involving humans and meta-analyses involving the association of risk factors and periodontal and dental implant outcomes were excluded. Meta-analyses other than pairwise were excluded. We defined different thematic areas in periodontology and implant dentistry to guide our systematic approach for selecting studies.

#### Periodontology

1) Local and systemic antibiotics for periodontal treatment
2) Regeneration procedures for infrabony defects (e.g., enamel matrix derivative, guided tissue regeneration, bone substitutes without membrane)
3) Periodontal plastic surgery (e.g., gingival recession procedures, soft tissue augmentation for the alveolar ridge)

#### Implant Dentistry

1) Nonsurgical treatment for mucositis and peri-implantitis
2) Surgical treatment for peri-implantitis
3) Different types (length/diameter) and surfaces of dental implants (e.g., survival of implant surface, treatment of peri-implant problems in surfaces)
4) Any kind of guided bone regeneration (e.g., external and internal sinus lift, vertical augmentation, site preservation)

Meta-analyses published in English containing at least 4 clinical trials were included. The minimum number of 4 trials was considered necessary for any trends over time to be detected.

### Search and Selection of Meta-analyses

In the PubMed database, we searched meta-analyses published between August 2015 and August 2020 by using specific keywords directly related to the thematic areas. We limited our search to more recent systematic reviews to allow a better comparison between the oldest trials and the meta-analysis pooled estimate. More recent reviews are more likely to include a larger number of trials in the meta-analyses. The search process was conducted independently and in duplicate by 2 authors (M.C.M., C.M.F.). For the detailed search strategy, see Appendix Table 1.

We selected the forest plots directly related to the primary outcomes of at least 4 trials. In case of no report of primary outcomes, we selected the forest plots with the greatest number of primary studies. If multiple selected forest plots had the same number of studies, we included all of them.

Two authors (M.C.M., C.M.F.) selected a sample of eligible studies and achieved good agreement (at least 80%), with the remainder selected by 1 reviewer (M.C.M.) (Shea et al. 2017).

### Data Extraction

We extracted the following information from the systematic reviews and meta-analyses: publication year, type of meta-analysis (Cochrane vs. non-Cochrane), type of trial (RCT and non-RCT, including design), continent of the first author, dental specialty (periodontology or implant dentistry), thematic area, journal impact factor, risk of bias (RoB) of primary studies (median, interquartile range [IQR]), type of outcome (binary, continuous), and type of synthesis (fixed or random effects). Two authors (M.C.M., C.M.F.) extracted data from a sample of eligible studies and achieved good agreement (at least 80%), with the remainder extracted by 1 reviewer (M.C.M.) (Shea et al. 2017).

### Data Analysis

We focused on meta-analyses that contain at least 4 trials and have a publication time span of at least 4 y, with the effect size measured as either a mean difference or a standardized mean difference. For standardization purposes and especially because the number of binary outcomes was limited, we analyzed continuous outcomes only. Statistically or clinically significant differences (as implied from the confidence and prediction intervals) between the first trial’s effect estimate and the meta-analysis pooled estimate are assumed to be a sign of novelty bias. Since reporting of PIs was rare, we calculated them by re-running all the included meta-analyses with the option to include PIs. To demonstrate this, we explored visually and statistically via logistic regression models if these differences are associated with various meta-analysis characteristics, such as the range of publication years, the number of trials, and the RoB (RoB of the first trial worse than the average RoB). We fitted 2 logistic regression models using the aforementioned trial characteristics as predictors to examine if there is an association between these characteristics and the odds of 1) the first trial’s estimate to be included in meta-analysis CI and 2) an overlap between the first trial’s CI and meta-analysis prediction interval (PI). The outcome in both scenarios is binary—such as overlap (no novelty bias suspected) and no overlap (novelty bias suspected)—between early estimates and pooled estimates CIs or overlap between the first trial’s CI and meta-analysis PI. The odds ratio (OR) is the effect measure used when fitting a logistic model, and higher odds with small *P* values would provide evidence against the null hypothesis. The PI shows the range of plausible values for future trials (Riley et al. 2011). In all analyses, we used a significance level of 5%. We used Wald’s statistic to test the significance of predictors. All analyses were conducted with Stata 16.1 (StataCorp) and R 3.6.1 (R Foundation for Statistical Computing).

## Results

### Study Characteristics

**Figure 1.**
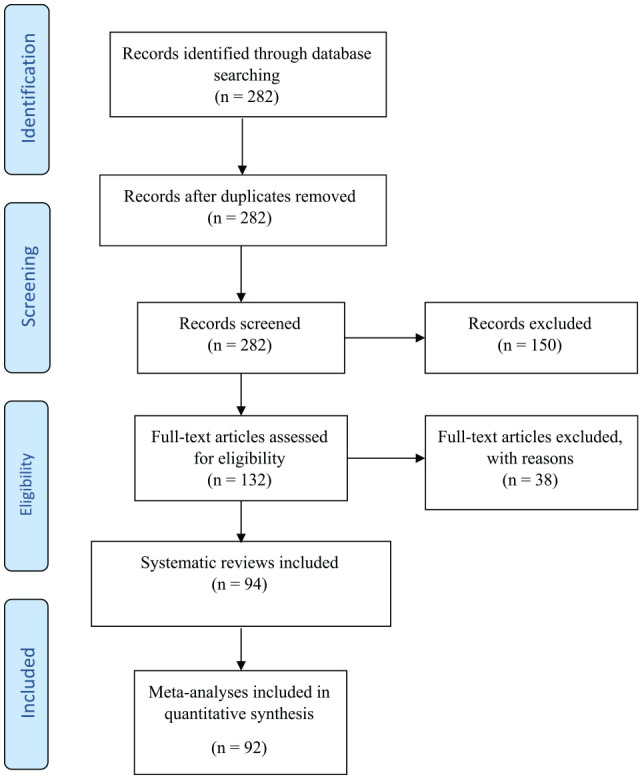
Flowchart of article selection.

The search initially retrieved 282 potential documents, and 94 systematic reviews were included, comprising 349 meta-analyses for potential assessment. Figure 1 presents the search and selection process. The full lists of included and excluded studies, with reasons for exclusion, are reported in the Appendix. Of the 349 meta-analyses fulfilling the initial eligibility criteria, 92 meta-analyses satisfied our inclusion criteria for novelty bias assessment. From the 94 systematic reviews, just 2 were Cochrane reviews, and the greatest proportion (*n* = 56, 60%) were published in the field of implant dentistry. Periodontal plastic surgery was the most prevalent topic (*n* = 28, 30%), and most systematic reviews were published in 2018 (*n* = 31, 33%). The first author of most systematic reviews (*n* = 34, 36%) was based in Europe, and the *Journal of Clinical Periodontology* had the greatest number (*n* = 10, 11%) of systematic reviews published. For systematic review characteristics, see Appendix Table 2. The meta-analyses had a median publication span of 8 y (IQR, 6 to 11; mean, 8.79; range, 4 to 22) and a median 6 studies (IQR, 5 to 9.25; mean, 7.61; range, 4 to 27).

The median sample size for the first trial was 30 (IQR, 23 to 44; mean, 41.98; range, 14 to 134). The median sample size for the pooled estimate was 282 (IQR, 197 to 469; mean, 369.86; range, 88 to 2,281).

### Data Analysis

Table 1 shows an overlap between the 95% CI of the first trial and 1) that of the meta-analysis pooled effect in 87% of the cases and 2) the 95% PI in 99% of the cases. In 58% of our meta-analyses, there was a significant pooled estimate, and in 70% the pooled estimate was smaller than the corresponding estimate of the first trial (in absolute values). The first trial’s estimate was included in meta-analysis confidence and prediction intervals at 52% and 92% of the cases, respectively.

**Table 1.** Observed and Expected (95% CI) Frequency of Different Cases.

	% (95% CI)	
	Yes	No
Overlap between meta-analysis		
Confidence interval and first trial’s confidence interval	87 (80 to 94)	13 (6 to 20)
Prediction interval and first trial’s confidence interval	99 (97 to 100)	1 (0 to 3)
Meta-analysis treatment estimates statistically significant	58 (48 to 68)	42 (32 to 52)
First trial’s		
Treatment estimate included in meta-analysis confidence interval	52 (42 to 62)	48 (38 to 58)
Absolute treatment estimate larger than meta-analysis absolute treatment estimate	70 (60 to 79)	30 (21 to 40)
Treatment estimate included in meta-analysis prediction interval	92 (87 to 98)	8 (2 to 13)

**Figure 2.**
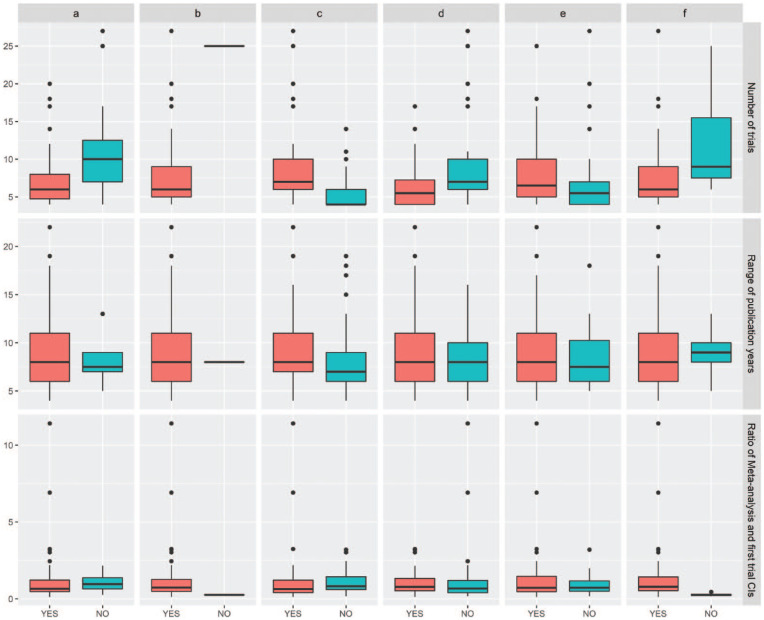
Box plots for the number of trials, the range of publication years, and the ratio between the range of the meta-analysis CI and the first trial’s CI, showing treatment effects when the first trial’s estimates fulfill (YES) or not (NO) the following criteria: (**a**) the CI overlaps with the meta-analysis CI, (**b**) the CI overlaps with the meta-analysis PI, (**c**) a statistically significant treatment estimate both in the first trial and meta-analysis results, (**d**) the treatment estimate is included in the meta-analysis CI, (**e**) the treatment estimate has a larger absolute value than the corresponding meta-analysis treatment estimate, and (**f**) the treatment estimate is included in the meta-analysis PI. Values are presented as limits of the box plot (line), interquartile range (box), and exaggerated effect estimates outside box plot limits (circles).

Differences in trial characteristics among the cases are presented in Figure 2. From the figure, we observe that the number of studies is higher when the meta-analysis treatment estimate is significant. Significant differences were not found for the range of the publication years and the ratio between the range of the meta-analysis CI and the range of the first trial’s CI.

**Figure 3.**
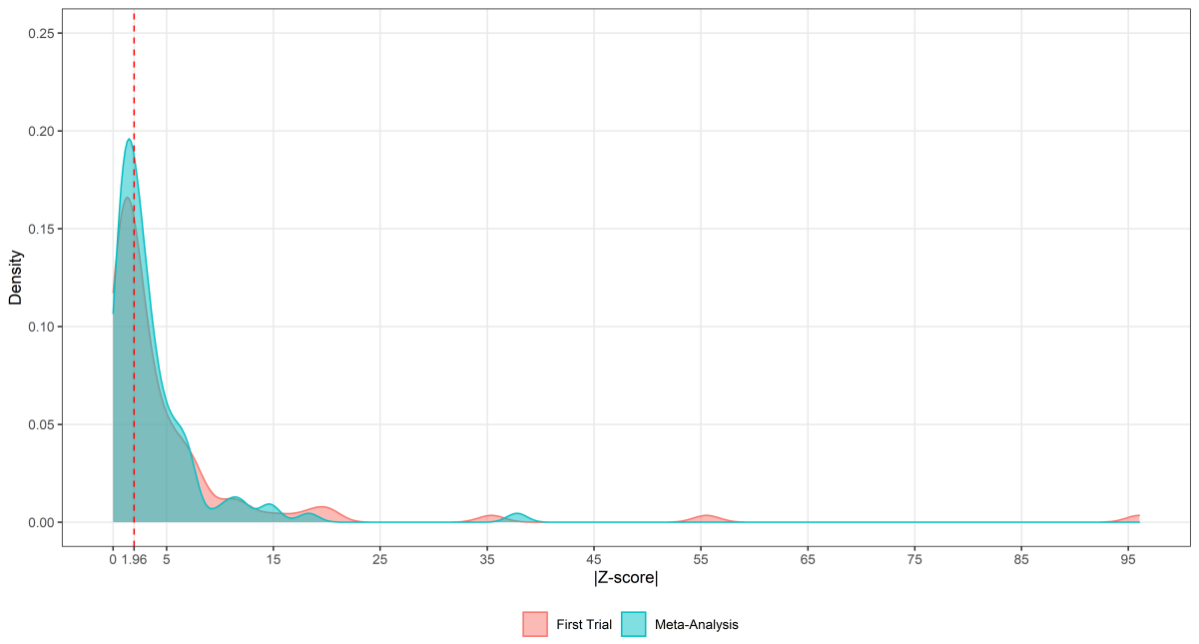
Density plot of the absolute *z* values of the Wald statistic between the first trial and the meta-analysis. The dotted line indicates the absolute *z* value with an equal likelihood of being observed in the meta-analysis and the first trial.

We computed Wald’s *z* values (effect over the standard error) for the effect size of the first trial and for the pooled effect. The density plot (Fig. 3) indicates that Wald’s absolute *z* values <1.96 are almost equally likely to be observed in the meta-analysis and the first trial. It should be noted that in the range between 0 and 5, it seems that the 2 distributions do not significantly differ.

We tested statistically whether the odds that the treatment effect estimates of the first trial to be included in the meta-analysis CI are associated with various meta-analysis characteristics and trial characteristics by applying a logistic regression model. We found that year of publication (OR, 1.06; 95% CI, 0.95 to 1.20) and RoB (OR, 1.50; 95% CI, 0.63 to 3.69) do not significantly affect these odds, but the odds of the 95% CI of the first trial’s effect overlapping the pooled 95% CI increases by 50% if the first study is at high RoB. The number of trials significantly influence the odds by reducing them 15% per 1 extra study in the meta-analysis (OR, 0.85; 95% CI, 0.73 to 0.96). The odds of observing an overlap between the first trial’s CI and the meta-analysis PI are significantly affected from only the number of trials, reducing the odds by 22% per extra study in the meta-analysis (OR, 0.78; 95% CI, 0.63 to 0.97; Table 2). It is likely that in most meta-analyses, we lack power to find significant differences between the effect estimate of the first trials and the meta-analysis pooled effect estimate. We found a median 6 studies, with 75% of the meta-analyses including up to 9 studies. In meta-analysis with a relatively large number of studies, novelty bias patterns are more evident. In both analyses, there was much uncertainty associated with the RoB.

**Table 2.** Logistic Regression Coefficients.

Predictor	Odds Ratio (95% CI)	*P* Value
Observe a treatment estimate of the first trial to be included in meta-analysis confidence interval		
Range of publication years	1.06 (0.95 to 1.20)	0.31
No. of trials	0.85 (0.73 to 0.96)	0.02
Risk of bias	1.50 (0.63 to 3.69)	0.37
Hosmer-Lemeshow goodness-of-fit test: χ^2^ = 11.25, *df* = 8, *P* = 0.19		
Observing an overlap between the first trial’s confidence interval and meta-analysis prediction interval^a^		
Range of publication years	0.85 (0.61 to 1.19)	0.34
No. of trials	0.78 (0.63 to 0.97)	0.03
Risk of bias	1.07 (0.04 to 27.34)	0.97
Hosmer-Lemeshow goodness of fit test: χ^2^ = 1.13, *df* = 8, *P* > 0.99		

a Estimates from penalized logistic regression.

## Discussion

The aim of this study was to assess the presence of novelty bias in periodontics and implant dentistry. We used as measures of novelty bias differences in the effect size between pooled meta-analysis treatment effects and first trial treatment effects as well as the degree of overlap in uncertainty measures, such as 95% confidence and prediction intervals. The PI—which is, unfortunately, not often used (Chiolero et al. 2012)—is a good indicator of the range of the treatment effect estimate of a future trial (Riley et al. 2011). The present findings suggest the possibility of novelty bias in the selected sample when comparing the first trial’s absolute treatment estimate and that of the meta-analysis pooled estimate. However, novelty bias seems to be fading when the basis of the assessment is the wider PI. Regression analyses indicated that among year of publication, RoB, and number of trials in the meta-analyses, only the last reached statistical significance.

Previous studies have reported signs of novelty bias (Barbui et al. 2004; Gehr et al. 2006; Salanti et al. 2010; Tinè et al. 2017; Cipriani et al. 2018). Salanti et al. (2010) identified novelty bias in cancer treatment using a meta-regression model. Similar patterns were observed for antidepressants (Cipriani et al. 2018) and for some lipid-lowering antiglaucoma drugs (Gehr et al. 2006). This tendency was confirmed in the present study where meta-analytic estimates were more conservative in absolute values; however, when the comparison moved from estimates to overlapping confidence and prediction intervals, novelty bias seemed to be waning. As the number of trials increased, the odds of nonoverlapping estimates and their range between the first study and the meta-analytic pooled estimate decrease, suggesting the presence of novelty bias. It is also reasonable to assume that as the number of studies increases, the estimates are more stable and that the confidence in the results increases.

In our sample, meta-analyses where the first trial treatment effects were not in the meta-analysis PI were identified in regeneration of periodontal tissues. For example, 2 meta-analyses focused on the treatment effect of the use of platelet concentrates as adjunctive for the treatment of gingival recessions (Li et al. 2019) and furcation defects (Panda et al. 2019). In the first case, the estimate of the first trial in the meta-analysis was −0.20, and the PI of the meta-analysis ranged from −0.18 to 0.74. In the meta-analysis on the treatment of furcation defects, the estimate of the first trial was 2.40, and the PI of the respective meta-analysis ranged from 0.56 to 2.11. Furthermore, 1 meta-analysis (da Rosa et al. 2018) assessed the efficacy of bioactive proteins for bone regeneration in implant-based rehabilitation. In this case, the first trial reported an estimate of –0.74 with the PI of the corresponding meta-analysis ranging from –0.61 to 0.07. The mechanism that may explain novelty bias is not fully clear. Novelty bias might be connected to vibrational effects due to flexible analyses and other types of bias, such as selective outcome reporting (Ioannidis 2008) and selection bias (Persaud and Heneghan 2021), when patients are selected on the basis of a favorable response to the proposed new therapy. Initial enthusiasm is not the only reason that we may observe a difference between first trial’s effect estimate and the pooled effect estimate. Other potential reasons include publication bias, RoB, and improvements in methodology. In our sample, the median publication time span is 8 y with most meta-analysis having a range <10 y. In such a relatively small time span, the impact of the aforementioned features on the difference between the first trial’s effect estimate and the pooled effect estimate may be limited. However, this is likely to be related with the type of intervention and the clinical research activity in the area. In addition, the number of trials in the meta-analyses is relatively small, suggesting in general low power in detecting novelty effects when the first trial estimate is compared with the PI.

Bias in trials is hard to quantify; however, there is empirical evidence suggesting measures that reduce bias in trials (Schulz et al. 1995; Savović et al. 2012; Savovic et al. 2018), such as randomization with allocation concealment and blinding, where feasible. It is not fully clear how novelty bias arises, but it has been suggested that it may be the result of a combination of biases observed during the design, conduct, analysis, and reporting of a trial (Ioannidis 2008; Persaud and Heneghan 2021). Therefore, abiding to solid trial methodology principles across all trial stages is expected on average to reduce novelty bias. Novelty bias is more likely when the evidence is limited and during the early period of introducing a new intervention. At those stages, the possibility of a false-positive result and/or publication based on the size and direction of the effect (publication bias) should not be ignored. From a practical perspective, clinicians should be aware to not easily adopt new treatment modalities with limited evidence.

### Strengths and Limitations

To our knowledge, this is one of the first studies in dentistry to address the topic of novelty bias. Although many meta-analyses likely lack power due to the limited number of trials, there was a substantial number of meta-analyses that allowed the analysis in various periodontology and implant dentistry topics. The use of prediction intervals is a new approach in assessing novelty bias, as it accounts for the uncertainty in the estimate of a future trial; this approach makes the results of this study more robust. Limitations of the study include reduced generalizability of our findings to other dental fields and the potential of selection bias, as only the meta-analyses published in English were selected. In addition, the endemic problem of the limited number of trials, which are usually small, and the relatively short range of publication years contribute to the uncertainty of the results. We also searched in only 1 major database, and some studies that could be important for comparisons may have been excluded. Nevertheless, we feel that a good number of meta-analyses were included that well represent the chosen field.

## Conclusion

The present findings suggest that there is some evidence of novelty bias in meta-analyses published in periodontology and implant dentistry. However, the often-limited number of trials across the various topics restricts the ability to fully assess novelty bias. Nevertheless, consumers of research should be aware that initially optimistic treatment effects may be influenced by novelty bias and can be refuted in future trials.

## Author Contributions

M.C. Menne, contributed to data acquisition, contributed to interpretation of the manuscript and critically revised the manuscript; G. Seitidis, contributed to the statistical analyses and interpretation, critically revised the manuscript; C.M. Faggion Jr, contributed to design, data acquisition, and interpretation, first drafted and critically revised the manuscript; D. Mavridis, contributed to design, statistical analyses, and interpretation, critically revised the manuscript; N. Pandis, contributed to conception, design, statistical analyses and interpretation, drafted and critically revised the manuscript. All authors gave final approval and agree to be accountable for all aspects of the work

## Supplemental Material

sj-docx-1-jdr-10.1177_00220345211025242 – Supplemental material for Early Optimistic Effect in Periodontology and Implant Dentistry Trials Click here for additional data file. Supplemental material, sj-docx-1-jdr-10.1177_00220345211025242 for Early Optimistic Effect in Periodontology and Implant Dentistry Trials by M.C. Menne, G. Seitidis, C.M. Faggion, D. Mavridis and N. Pandis in Journal of Dental Research
