# Supplementary material for: Early Optimistic Effect in Periodontology and Implant Dentistry Trials
Source: J Dent Res. 2021 Jul 8;101(1):30–6. doi: 10.1177/00220345211025242 (PMC8721552; doi:10.1177/00220345211025242)
Supplement: sj-docx-1-jdr-10.1177_00220345211025242 – Supplemental material for Early Optimistic Effect in Periodontology and Implant Dentistry Trials [file sj-docx-1-jdr-10.1177_00220345211025242.docx]

**Title: Early optimistic effect in periodontology and implant dentistry trials**

**Authors:** Max Clemens Menne^1^, Georgios Seitidis^2^, Clovis Mariano Faggion Jr^1^, Dimitris Mavridis^2^, Nikolaos Pandis^3^

^1^ Department of Periodontology and Operative Dentistry, Faculty of Dentistry, University Hospital Münster, Münster, Germany

^2^ Department of Primary Education, School of Education, University of Ioannina, Ioannina, Greece

^3^ Department of Orthodontics and Dentofacial Orthopedics, Dental School/Medical Faculty, University of Bern, Switzerland

**Corresponding author:** Clovis Mariano Faggion Jr, Faculty of Dentistry, University Hospital Münster, Waldeyerstraße 30, 48149 Münster, Germany. Tel +49 (0)251 / 83-47061, Fax +49 (0)251 / 83-47134, Email: [clovisfaggion@yahoo.com](mailto:clovisfaggion@yahoo.com)

**Supplementary file**

Table of contents

[Table S1. Literature search applied to the PubMed database 2](#_Toc70599432)

[Table S2. Characteristics of systematic reviews, meta-analyses and trials 3](#_Toc70599433)

[List S3. Included SRs 6](#_Toc70599434)

[List S4. Excluded SRs after assessment of the title and abstract 15](#_Toc70599435)

[List S5. Excluded SRs after assessment of the full text 30](#_Toc70599436)

##

## Table S1. Literature search applied to the PubMed database

#12Search: #8 AND #9 Filters: Meta-Analysis, in the last 5 years 282 (All fields)

#11Search: #8 AND #9 Filters: Meta-Analysis 556

#10Search: #8 AND #9 35,599

#9Search: Therapeutic OR Therapy OR Therapies OR Treatment OR Treatments 11,689,471

#8Search: #1 OR #2 OR #3 OR #4 OR #5 OR #6 OR #7 51,509

#7Search: peri-implantitis OR periimplantitis OR "peri implantitis" 2,652

#6Search: "dental implants" 29,129

#5Search: antimicrobials AND periodont* 11,370

#4Search: "gingival recession" 4,472

#3Search: "periodontal regeneration" 1,806

#2Search: "guided tissue regeneration" 6,398

#1Search: antibiotics AND periodont* 6,283

## Table S2. Characteristics of systematic reviews, meta-analyses and trials

|  | Total (N= 94) |
| --- | --- |
| Characteristics of systematic reviews | **n (%)** |
| Type of Systematic Review |  |
| Non-Cochrane Review | 92 (98%) |
| Cochrane Review | 2 (2%) |
| Dental Discipline |  |
| Implantology | 56 (60%) |
| Periodontology | 38 (40%) |
| Main Topic of the Systematic Review |  |
| Periodontal Plastic Surgery | 28 (30%) |
| Bone Augmentation Procedures | 20 (21%) |
| Implant Characteristics | 15 (16%) |
| Non-Surgical Treatment of Peri-Implantitis | 9 (10%) |
| Implant Characteristics and Bone Augmentation Procedures | 8 (9%) |
| Regeneration Procedures for Infrabony Defects | 5 (5%) |
| Local Antibiotics for Periodontal Therapy | 3 (3%) |
| Regeneration Procedures for Infrabony Defects and Periodontal Plastic Surgery | 2 (2%) |
| Non-Surgical and Surgical Treatment of Peri-Implantitis | 2 (2%) |
| Surgical Treatment of Peri-Implantitis | 1 (1%) |
| Local Antibiotics for Periodontal Therapy and Periodontal Plastic Surgery | 1 (1%) |
| Year of publication |  |
| 2015 | 9 (10%) |
| 2016 | 17 (18%) |
| 2017 | 8 (9%) |
| 2018 | 31 (33%) |
| 2019 | 22 (23%) |
| 2020 | 7 (7%) |
| Continent of the first author |  |
| Europe | 34 (36%) |
| Asia | 30 (32%) |
| America | 26 (28%) |
| Africa | 2 (2%) |
| Australia | 2 (2%) |
|  |  |
| Impact-Factor |  |
| Median (IQR) | 2,426 (1,937) |
| Citations |  |
| Median (IQR) | 15 (28) |
|  | **Total (N = 349)** |
| Characteristics of meta-analyses | **n (%)** |
| Meta-Analysis Method |  |
| Fixed | 81 (23,2%) |
| Random | 263 (75,4%) |
| Unclear | 5 (1,4%) |
|  | **Total (N = 1748)** |
| Characteristics of trials | **n (%)** |
| Types of Included RCTs |  |
| Total | **1294 (74%)** |
| Parallel | 362 (21%) |
| Split-Mouth | 315 (18%) |
| Parallel and Split-Mouth | 2 (0%) |
| Other | 3 (0%) |
| Unspecified | 612 (35%) |
| Types of Included Non-RCTs |  |
| Total | **365 (21%)** |
| CCT | 33 (2%) |
| Case Series | 64 (4%) |
| Prospective Trial | 199 (11%) |
| Retrospective Trial | 47 (3%) |
| Cohort Study | 5 (0%) |
| Pilot Study | 10 (1%) |
| Unspecified | 7 (0%) |
| No information about randomization |  |
| No Information about Randomization and Design | **89 (5%)** |
| Risk of Bias in Included Studies |  |
| Low | 233 (13%) |
| Unclear | 406 (23%) |
| High | 531 (30%) |
| Not Reported | 397 (23%) |
| Not Applicable | 181 (10%) |
| Outcomes of Included Studies |  |
| Binary | 582 (33%) |
| Continuous | 1033 (59%) |
| Not Reported | 133 (8%) |

## List S3. Included SRs

Abdalla, R. I. B., A. Y. Alqutaibi, and A. Kaddah. “Does the Adjunctive Use of Platelet-Rich Plasma to Bone Graft during Sinus Augmentation Reduce Implant Failure and Complication? Systematic Review and Meta-Analysis.” *Quintessence Int* 49, no. 2 (2018): 139–46. <https://doi.org/10.3290/j.qi.a39616>.

Akram, Z., T. Hyder, N. Al-Hamoudi, M. S. Binshabaib, S. S. Alharthi, and A. Hanif. “Efficacy of Photodynamic Therapy versus Antibiotics as an Adjunct to Scaling and Root Planing in the Treatment of Periodontitis: A Systematic Review and Meta-Analysis.” *Photodiagnosis Photodyn Ther* 19 (September 2017): 86–92. <https://doi.org/10.1016/j.pdpdt.2017.05.007>.

Akram, Z., S. S. Shafqat, M. O. Niaz, A. Raza, and M. Naseem. “Clinical Efficacy of Photodynamic Therapy and Laser Irradiation as an Adjunct to Open Flap Debridement in the Treatment of Chronic Periodontitis: A Systematic Review and Meta-Analysis.” *Photodermatol Photoimmunol Photomed* 36, no. 1 (January 2020): 3–13. <https://doi.org/10.1111/phpp.12499>.

Akram, Z., F. Vohra, and F. Javed. “Efficacy of Statin Delivery as an Adjunct to Scaling and Root Planing in the Treatment of Chronic Periodontitis: A Meta-Analysis.” *J Investig Clin Dent* 9, no. 2 (May 2018): e12304. <https://doi.org/10.1111/jicd.12304>.

Akram, Z., F. Vohra, and F. Javed. “Low-Level Laser Therapy as an Adjunct to Connective Tissue Graft Procedure in the Treatment of Gingival Recession Defects: A Systematic Review and Meta-Analysis.” *J Esthet Restor Dent* 30, no. 4 (July 2018): 299–306. <https://doi.org/10.1111/jerd.12377>.

Altaib, F. H., A. Y. Alqutaibi, A. Al-Fahd, and S. Eid. “Short Dental Implant as Alternative to Long Implant with Bone Augmentation of the Atrophic Posterior Ridge: A Systematic Review and Meta-Analysis of RCTs.” *Quintessence Int* 50, no. 8 (2019): 636–50. <https://doi.org/10.3290/j.qi.a42948>.

Altuna, P., E. Lucas-Taulé, J. Gargallo-Albiol, O. Figueras-Álvarez, F. Hernández-Alfaro, and J. Nart. “Clinical Evidence on Titanium-Zirconium Dental Implants: A Systematic Review and Meta-Analysis.” *Int J Oral Maxillofac Surg* 45, no. 7 (July 2016): 842–50. <https://doi.org/10.1016/j.ijom.2016.01.004>.

Aludden, H., A. Mordenfeld, M. Hallman, A. E. Christensen, and T. Starch-Jensen. “Osteotome-Mediated Sinus Floor Elevation With or Without a Grafting Material: A Systematic Review and Meta-Analysis of Long-Term Studies (≥5-Years).” *Implant Dent* 27, no. 4 (August 2018): 488–97. <https://doi.org/10.1097/id.0000000000000798>.

Apparaju, V., S. K. Vaddamanu, R. Vyas, S. Vishwanath, V. Gurumurthy, and M. A. Kanji. “Is Balloon-Assisted Maxillary Sinus Floor Augmentation before Dental Implant Safe and Promising? A Systematic Review and Meta-Analysis.” *Niger J Clin Pract* 23, no. 3 (March 2020): 275–83. <https://doi.org/10.4103/njcp.njcp_238_19>.

Aslam, A., and B. Ahmed. “Platform-Switching to Preserve Peri-Implant Bone: A Meta-Analysis.” *J Coll Physicians Surg Pak* 26, no. 4 (April 2016): 315–19.

Atieh, M. A., and N. H. M. Alsabeeha. “Soft Tissue Changes after Connective Tissue Grafts around Immediately Placed and Restored Dental Implants in the Esthetic Zone: A Systematic Review and Meta-Analysis.” *J Esthet Restor Dent* 32, no. 3 (April 2020): 280–90. <https://doi.org/10.1111/jerd.12538>.

Atieh, M. A., N. Alsabeeha, A. Tawse-Smith, and A. G. Payne. “Xenogeneic Collagen Matrix for Periodontal Plastic Surgery Procedures: A Systematic Review and Meta-Analysis.” *J Periodontal Res* 51, no. 4 (August 2016): 438–52. <https://doi.org/10.1111/jre.12333>.

Barootchi, S., A. Ravidà, L. Tavelli, and H. L. Wang. “Nonsurgical Treatment for Peri-Implant Mucositis: A Systematic Review and Meta-Analysis.” *Int J Oral Implantol (Berl)* 13, no. 2 (2020): 123–39.

Barootchi, S., L. Tavelli, A. Ravidà, C. W. Wang, and H. L. Wang. “Effect of EDTA Root Conditioning on the Outcome of Coronally Advanced Flap with Connective Tissue Graft: A Systematic Review and Meta-Analysis.” *Clin Oral Investig* 22, no. 8 (November 2018): 2727–41. <https://doi.org/10.1007/s00784-018-2635-3>.

Behdin, S., A. Monje, G. H. Lin, B. Edwards, A. Othman, and H. L. Wang. “Effectiveness of Laser Application for Periodontal Surgical Therapy: Systematic Review and Meta-Analysis.” *J Periodontol* 86, no. 12 (December 2015): 1352–63. <https://doi.org/10.1902/jop.2015.150212>.

Bertl, K., M. Melchard, N. Pandis, M. Müller-Kern, and A. Stavropoulos. “Soft Tissue Substitutes in Non-Root Coverage Procedures: A Systematic Review and Meta-Analysis.” *Clin Oral Investig* 21, no. 2 (March 2017): 505–18. <https://doi.org/10.1007/s00784-016-2044-4>.

Braun, R. S., L. Chambrone, and I. Khouly. “Prophylactic Antibiotic Regimens in Dental Implant Failure: A Systematic Review and Meta-Analysis.” *J Am Dent Assoc* 150, no. 6 (June 2019): e61–91. <https://doi.org/10.1016/j.adaj.2018.10.015>.

Camps-Font, O., G. Burgueño-Barris, R. Figueiredo, R. E. Jung, C. Gay-Escoda, and E. Valmaseda-Castellón. “Interventions for Dental Implant Placement in Atrophic Edentulous Mandibles: Vertical Bone Augmentation and Alternative Treatments. A Meta-Analysis of Randomized Clinical Trials.” *J Periodontol* 87, no. 12 (December 2016): 1444–57. <https://doi.org/10.1902/jop.2016.160226>.

Camps-Font, O., C. Caro-Bonfill, MÀ Sánchez-Garcés, and C. Gay-Escoda. “Periodontal Regenerative Therapy for Preventing Bone Defects Distal to Mandibular Second Molars After Surgical Removal of Impacted Third Molars: A Systematic Review and Meta-Analysis of Randomized Clinical Trials.” *J Oral Maxillofac Surg* 76, no. 12 (December 2018): 2482–2514. <https://doi.org/10.1016/j.joms.2018.07.025>.

Canullo, L., M. Menini, G. Santori, M. Rakic, A. Sculean, and P. Pesce. “Titanium Abutment Surface Modifications and Peri-Implant Tissue Behavior: A Systematic Review and Meta-Analysis.” *Clin Oral Investig* 24, no. 3 (March 2020): 1113–24. <https://doi.org/10.1007/s00784-020-03210-x>.

Castro, A. B., N. Meschi, A. Temmerman, N. Pinto, P. Lambrechts, W. Teughels, and M. Quirynen. “Regenerative Potential of Leucocyte- and Platelet-Rich Fibrin. Part A: Intra-Bony Defects, Furcation Defects and Periodontal Plastic Surgery. A Systematic Review and Meta-Analysis.” *J Clin Periodontol* 44, no. 1 (January 2017): 67–82. <https://doi.org/10.1111/jcpe.12643>.

Chambrone, L., M. A. Salinas Ortega, F. Sukekava, R. Rotundo, Z. Kalemaj, J. Buti, and G. P. Pini Prato. “Root Coverage Procedures for Treating Localised and Multiple Recession-Type Defects.” *Cochrane Database Syst Rev* 10, no. 10 (October 2, 2018): Cd007161. <https://doi.org/10.1002/14651858.CD007161.pub3>.

Chen, M. H., and J. Y. Shi. “Clinical and Radiological Outcomes of Implants in Osteotome Sinus Floor Elevation with and without Grafting: A Systematic Review and a Meta-Analysis.” *J Prosthodont* 27, no. 5 (June 2018): 394–401. <https://doi.org/10.1111/jopr.12576>.

Chen, Y., C. Liu, X. Chen, and A. Mo. “Clinical Evidence of Photobiomodulation Therapy (PBMT) on Implant Stability and Success: A Systematic Review and Meta-Analysis.” *BMC Oral Health* 19, no. 1 (May 7, 2019): 77. <https://doi.org/10.1186/s12903-019-0779-4>.

Chen, Z., Y. Zhang, J. Li, H. L. Wang, and H. Yu. “Influence of Laser-Microtextured Surface Collar on Marginal Bone Loss and Peri-Implant Soft Tissue Response: A Systematic Review and Meta-Analysis.” *J Periodontol* 88, no. 7 (July 2017): 651–62. <https://doi.org/10.1902/jop.2017.160805>.

Clementini, M., A. Ambrosi, V. Cicciarelli, V. De Risi, and M. de Sanctis. “Clinical Performance of Minimally Invasive Periodontal Surgery in the Treatment of Infrabony Defects: Systematic Review and Meta-Analysis.” *J Clin Periodontol* 46, no. 12 (December 2019): 1236–53. <https://doi.org/10.1111/jcpe.13201>.

Corbella, S., S. Taschieri, R. Weinstein, and M. Del Fabbro. “Histomorphometric Outcomes after Lateral Sinus Floor Elevation Procedure: A Systematic Review of the Literature and Meta-Analysis.” *Clin Oral Implants Res* 27, no. 9 (September 2016): 1106–22. <https://doi.org/10.1111/clr.12702>.

Cruz, R. S., C. A. A. Lemos, V. E. S. Batista, Hffe Oliveira, J. M. L. Gomes, E. P. Pellizzer, and F. R. Verri. “Short Implants versus Longer Implants with Maxillary Sinus Lift. A Systematic Review and Meta-Analysis.” *Braz Oral Res* 32 (2018): e86. <https://doi.org/10.1590/1807-3107bor-2018.vol32.0086>.

Dai, A., J. P. Huang, P. H. Ding, and L. L. Chen. “Long-Term Stability of Root Coverage Procedures for Single Gingival Recessions: A Systematic Review and Meta-Analysis.” *J Clin Periodontol* 46, no. 5 (May 2019): 572–85. <https://doi.org/10.1111/jcpe.13106>.

Del Fabbro, M., L. Karanxha, S. Panda, C. Bucchi, J. Nadathur Doraiswamy, M. Sankari, S. Ramamoorthi, S. Varghese, and S. Taschieri. “Autologous Platelet Concentrates for Treating Periodontal Infrabony Defects.” *Cochrane Database Syst Rev* 11, no. 11 (November 26, 2018): Cd011423. <https://doi.org/10.1002/14651858.CD011423.pub2>.

Deng, Y., X. Zhu, J. Yang, H. Jiang, and P. Yan. “The Effect of Regeneration Techniques on Periapical Surgery With Different Protocols for Different Lesion Types: A Meta-Analysis.” *J Oral Maxillofac Surg* 74, no. 2 (February 2016): 239–46. <https://doi.org/10.1016/j.joms.2015.10.007>.

Elnayef, B., C. Porta, F. Suárez-López Del Amo, L. Mordini, J. Gargallo-Albiol, and F. Hernández-Alfaro. “The Fate of Lateral Ridge Augmentation: A Systematic Review and Meta-Analysis.” *Int J Oral Maxillofac Implants* 33, no. 3 (June 2018): 622–35. <https://doi.org/10.11607/jomi.6290>.

Fan, T., Y. Li, W. W. Deng, T. Wu, and W. Zhang. “Short Implants (5 to 8 Mm) Versus Longer Implants (>8 Mm) with Sinus Lifting in Atrophic Posterior Maxilla: A Meta-Analysis of RCTs.” *Clin Implant Dent Relat Res* 19, no. 1 (February 2017): 207–15. <https://doi.org/10.1111/cid.12432>.

Franchini, M., M. Cruciani, C. Mengoli, F. Masiello, G. Marano, E. D’Aloja, C. Dell’Aringa, et al. “The Use of Platelet-Rich Plasma in Oral Surgery: A Systematic Review and Meta-Analysis.” *Blood Transfus* 17, no. 5 (September 2019): 357–67. <https://doi.org/10.2450/2019.0177-19>.

Gao, J., S. Yu, X. Zhu, Y. Yan, Y. Zhang, and D. Pei. “Does Probiotic Lactobacillus Have an Adjunctive Effect in the Nonsurgical Treatment of Peri-Implant Diseases? A Systematic Review and Meta-Analysis.” *J Evid Based Dent Pract* 20, no. 1 (March 2020): 101398. <https://doi.org/10.1016/j.jebdp.2020.101398>.

Gargallo-Albiol, J., S. Barootchi, L. Tavelli, and H. L. Wang. “Efficacy of Xenogeneic Collagen Matrix to Augment Peri-Implant Soft Tissue Thickness Compared to Autogenous Connective Tissue Graft: A Systematic Review and Meta-Analysis.” *Int J Oral Maxillofac Implants* 34, no. 5 (October 2019): 1059–69. <https://doi.org/10.11607/jomi.7497>.

Hameed, M. H., M. Gul, R. Ghafoor, and F. R. Khan. “Vertical Ridge Gain with Various Bone Augmentation Techniques: A Systematic Review and Meta-Analysis.” *J Prosthodont* 28, no. 4 (April 2019): 421–27. <https://doi.org/10.1111/jopr.13028>.

Haro Adánez, M., H. Nishihara, and W. Att. “A Systematic Review and Meta-Analysis on the Clinical Outcome of Zirconia Implant-Restoration Complex.” *J Prosthodont Res* 62, no. 4 (October 2018): 397–406. <https://doi.org/10.1016/j.jpor.2018.04.007>.

Hou, X., J. Yuan, A. Aisaiti, Y. Liu, and J. Zhao. “The Effect of Platelet-Rich Plasma on Clinical Outcomes of the Surgical Treatment of Periodontal Intrabony Defects: A Systematic Review and Meta-Analysis.” *BMC Oral Health* 16, no. 1 (August 17, 2016): 71. <https://doi.org/10.1186/s12903-016-0261-5>.

Khouly, I., R. S. Braun, and L. Chambrone. “Antibiotic Prophylaxis May Not Be Indicated for Prevention of Dental Implant Infections in Healthy Patients. A Systematic Review and Meta-Analysis.” *Clin Oral Investig* 23, no. 4 (April 2019): 1525–53. <https://doi.org/10.1007/s00784-018-2762-x>.

Koodaryan, R., and A. Hafezeqoran. “Evaluation of Implant Collar Surfaces for Marginal Bone Loss: A Systematic Review and Meta-Analysis.” *Biomed Res Int* 2016 (2016): 4987526. <https://doi.org/10.1155/2016/4987526>.

Lee, C. T., L. Hum, and Y. W. Chen. “The Effect of Regenerative Periodontal Therapy in Preventing Periodontal Defects after the Extraction of Third Molars: A Systematic Review and Meta-Analysis.” *J Am Dent Assoc* 147, no. 9 (September 2016): 709-719.e4. <https://doi.org/10.1016/j.adaj.2016.03.005>.

Lee, J., J. B. Lee, K. T. Koo, Y. J. Seol, and Y. M. Lee. “Flap Management in Alveolar Ridge Preservation: A Systematic Review and Meta-Analysis.” *Int J Oral Maxillofac Implants* 33, no. 3 (June 2018): 613–21. <https://doi.org/10.11607/jomi.6368>.

Lemos, C. A., M. L. Ferro-Alves, R. Okamoto, M. R. Mendonça, and E. P. Pellizzer. “Short Dental Implants versus Standard Dental Implants Placed in the Posterior Jaws: A Systematic Review and Meta-Analysis.” *J Dent* 47 (April 2016): 8–17. <https://doi.org/10.1016/j.jdent.2016.01.005>.

Lemos, C. A., C. C. Mello, D. M. dos Santos, F. R. Verri, M. C. Goiato, and E. P. Pellizzer. “Effects of Platelet-Rich Plasma in Association with Bone Grafts in Maxillary Sinus Augmentation: A Systematic Review and Meta-Analysis.” *Int J Oral Maxillofac Surg* 45, no. 4 (April 2016): 517–25. <https://doi.org/10.1016/j.ijom.2015.07.012>.

Rosa, W. L. O. da, T. M. da Silva, A. F. da Silva, and E. Piva. “Bioactive Treatments in Bone Grafts for Implant-Based Rehabilitation: Systematic Review and Meta-Analysis.” *Clin Implant Dent Relat Res* 20, no. 2 (April 2018): 251–60. <https://doi.org/10.1111/cid.12552>.

Sousa, C. A. de, C. A. A. Lemos, J. F. Santiago-Júnior, L. P. Faverani, and E. P. Pellizzer. “Bone Augmentation Using Autogenous Bone versus Biomaterial in the Posterior Region of Atrophic Mandibles: A Systematic Review and Meta-Analysis.” *J Dent* 76 (September 2018): 1–8. <https://doi.org/10.1016/j.jdent.2018.06.014>.

Li, F., P. Jiang, J. Pan, C. Liu, and L. Zheng. “Synergistic Application of Platelet-Rich Fibrin and 1% Alendronate in Periodontal Bone Regeneration: A Meta-Analysis.” *Biomed Res Int* 2019 (2019): 9148183. <https://doi.org/10.1155/2019/9148183>.

Li, F., F. Yu, X. Xu, C. Li, D. Huang, X. Zhou, L. Ye, and L. Zheng. “Evaluation of Recombinant Human FGF-2 and PDGF-BB in Periodontal Regeneration: A Systematic Review and Meta-Analysis.” *Sci Rep* 7, no. 1 (March 6, 2017): 65. <https://doi.org/10.1038/s41598-017-00113-y>.

Li, R., Y. Liu, T. Xu, H. Zhao, J. Hou, Y. Wu, and D. Zhang. “The Additional Effect of Autologous Platelet Concentrates to Coronally Advanced Flap in the Treatment of Gingival Recessions: A Systematic Review and Meta-Analysis.” *Biomed Res Int* 2019 (2019): 2587245. <https://doi.org/10.1155/2019/2587245>.

Lin, C. Y., Z. Chen, W. L. Pan, and H. L. Wang. “Impact of Timing on Soft Tissue Augmentation during Implant Treatment: A Systematic Review and Meta-Analysis.” *Clin Oral Implants Res* 29, no. 5 (May 2018): 508–21. <https://doi.org/10.1111/clr.13148>.

Lin, C. Y., Z. Chen, W. L. Pan, and H. L. Wang. “The Effect of Supportive Care in Preventing Peri-Implant Diseases and Implant Loss: A Systematic Review and Meta-Analysis.” *Clin Oral Implants Res* 30, no. 8 (August 2019): 714–24. <https://doi.org/10.1111/clr.13496>.

Lozano-Carrascal, N., A. Anglada-Bosqued, O. Salomó-Coll, F. Hernández-Alfaro, H. L. Wang, and J. Gargallo-Albiol. “Short Implants (<8mm) versus Longer Implants (≥8mm) with Lateral Sinus Floor Augmentation in Posterior Atrophic Maxilla: A Meta-Analysis of RCT`s in Humans.” *Med Oral Patol Oral Cir Bucal* 25, no. 2 (March 1, 2020): e168–79. <https://doi.org/10.4317/medoral.23248>.

Miguita, L., A. Mantesso, C. M. Pannuti, and M. C. Z. Deboni. “Can Stem Cells Enhance Bone Formation in the Human Edentulous Alveolar Ridge? A Systematic Review and Meta-Analysis.” *Cell Tissue Bank* 18, no. 2 (June 2017): 217–28. <https://doi.org/10.1007/s10561-017-9612-y>.

Miron, R. J., V. Moraschini, M. Del Fabbro, A. Piattelli, M. Fujioka-Kobayashi, Y. Zhang, N. Saulacic, et al. “Use of Platelet-Rich Fibrin for the Treatment of Gingival Recessions: A Systematic Review and Meta-Analysis.” *Clin Oral Investig* 24, no. 8 (August 2020): 2543–57. <https://doi.org/10.1007/s00784-020-03400-7>.

Moraschini, V., D. C. F. de Almeida, S. Sartoretto, H. Bailly Guimarães, I. Chaves Cavalcante, and M. Diuana Calasans-Maia. “Clinical Efficacy of Xenogeneic Collagen Matrix in the Treatment of Gingival Recession: A Systematic Review and Meta-Analysis.” *Acta Odontol Scand* 77, no. 6 (August 2019): 457–67. <https://doi.org/10.1080/00016357.2019.1588372>.

Moraschini, V., and S. Barboza Edos. “Use of Platelet-Rich Fibrin Membrane in the Treatment of Gingival Recession: A Systematic Review and Meta-Analysis.” *J Periodontol* 87, no. 3 (March 2016): 281–90. <https://doi.org/10.1902/jop.2015.150420>.

Naenni, N., H. C. Lim, S. N. Papageorgiou, and C. H. F. Hämmerle. “Efficacy of Lateral Bone Augmentation Prior to Implant Placement: A Systematic Review and Meta-Analysis.” *J Clin Periodontol* 46 Suppl 21 (June 2019): 287–306. <https://doi.org/10.1111/jcpe.13052>.

Nielsen, H. B., S. Schou, F. Isidor, A. E. Christensen, and T. Starch-Jensen. “Short Implants (≤8mm) Compared to Standard Length Implants (>8mm) in Conjunction with Maxillary Sinus Floor Augmentation: A Systematic Review and Meta-Analysis.” *Int J Oral Maxillofac Surg* 48, no. 2 (February 2019): 239–49. <https://doi.org/10.1016/j.ijom.2018.05.010>.

Niño-Sandoval, T. C., B. C. Vasconcelos, D. Moraes SL, A. Lemos CA, and E. P. Pellizzer. “Efficacy of Stem Cells in Maxillary Sinus Floor Augmentation: Systematic Review and Meta-Analysis.” *Int J Oral Maxillofac Surg* 48, no. 10 (October 2019): 1355–66. <https://doi.org/10.1016/j.ijom.2018.04.022>.

Panda, S., L. Karanxha, F. Goker, A. Satpathy, S. Taschieri, L. Francetti, A. C. Das, M. Kumar, S. Panda, and M. D. Fabbro. “Autologous Platelet Concentrates in Treatment of Furcation Defects-A Systematic Review and Meta-Analysis.” *Int J Mol Sci* 20, no. 6 (March 17, 2019). <https://doi.org/10.3390/ijms20061347>.

Parize, H. N., L. O. L. Bohner, L. T. Gama, A. L. Porporatti, L. A. M. Mezzomo, W. C. Martin, and Tmsv Gonçalves. “Narrow-Diameter Implants in the Anterior Region: A Meta-Analysis.” *Int J Oral Maxillofac Implants* 34, no. 6 (December 2019): 1347–58. <https://doi.org/10.11607/jomi.7526>.

Pérez-González, F., P. Molinero-Mourelle, L. Sánchez-Labrador, L. M. Sáez-Alcaide, A. Limones, J. Cortés-Bretón Brinkmann, and J. López-Quiles. “Assessment of Clinical Outcomes and Histomorphometric Findings in Alveolar Ridge Augmentation Procedures with Allogeneic Bone Block Grafts: A Systematic Review and Meta-Analysis.” *Med Oral Patol Oral Cir Bucal* 25, no. 2 (March 1, 2020): e291–98. <https://doi.org/10.4317/medoral.23353>.

Raghoebar, G. M., P. Onclin, G. C. Boven, A. Vissink, and H. J. A. Meijer. “Long-Term Effectiveness of Maxillary Sinus Floor Augmentation: A Systematic Review and Meta-Analysis.” *J Clin Periodontol* 46 Suppl 21 (June 2019): 307–18. <https://doi.org/10.1111/jcpe.13055>.

Ramanauskaite, A., P. Daugela, and G. Juodzbalys. “Treatment of Peri-Implantitis: Meta-Analysis of Findings in a Systematic Literature Review and Novel Protocol Proposal.” *Quintessence Int* 47, no. 5 (2016): 379–93. <https://doi.org/10.3290/j.qi.a35131>.

Ravidà, A., I. C. Wang, S. Barootchi, H. Askar, L. Tavelli, J. Gargallo-Albiol, and H. L. Wang. “Meta-Analysis of Randomized Clinical Trials Comparing Clinical and Patient-Reported Outcomes between Extra-Short (≤6 Mm) and Longer (≥10 Mm) Implants.” *J Clin Periodontol* 46, no. 1 (January 2019): 118–42. <https://doi.org/10.1111/jcpe.13026>.

Ravidà, A., I. C. Wang, G. Sammartino, S. Barootchi, M. Tattan, G. Troiano, L. Laino, G. Marenzi, U. Covani, and H. L. Wang. “Prosthetic Rehabilitation of the Posterior Atrophic Maxilla, Short (≤6 Mm) or Long (≥10 Mm) Dental Implants? A Systematic Review, Meta-Analysis, and Trial Sequential Analysis: Naples Consensus Report Working Group A.” *Implant Dent* 28, no. 6 (December 2019): 590–602. <https://doi.org/10.1097/id.0000000000000919>.

Roccuzzo, M., D. M. Layton, A. Roccuzzo, and L. J. Heitz-Mayfield. “Clinical Outcomes of Peri-Implantitis Treatment and Supportive Care: A Systematic Review.” *Clin Oral Implants Res* 29 Suppl 16 (October 2018): 331–50. <https://doi.org/10.1111/clr.13287>.

Roehling, S., K. A. Schlegel, H. Woelfler, and M. Gahlert. “Performance and Outcome of Zirconia Dental Implants in Clinical Studies: A Meta-Analysis.” *Clin Oral Implants Res* 29 Suppl 16 (October 2018): 135–53. <https://doi.org/10.1111/clr.13352>.

Roselló-Camps, À, A. Monje, G. H. Lin, V. Khoshkam, M. Chávez-Gatty, H. L. Wang, J. Gargallo-Albiol, and F. Hernandez-Alfaro. “Platelet-Rich Plasma for Periodontal Regeneration in the Treatment of Intrabony Defects: A Meta-Analysis on Prospective Clinical Trials.” *Oral Surg Oral Med Oral Pathol Oral Radiol* 120, no. 5 (November 2015): 562–74. <https://doi.org/10.1016/j.oooo.2015.06.035>.

Rovai, E. S., L. M. B. Ambrosio, C. M. R. Morillo, C. C. Villar, M. Holzhausen, M. P. Santamaria, and C. M. Pannuti. “Root Coverage Procedures in Noncarious Cervical Lesions With and Without Restoration: A Systematic Review and Meta-Analysis.” *Int J Periodontics Restorative Dent* 40, no. 3 (June 2020): e127–35. <https://doi.org/10.11607/prd.4284>.

Santiago, J. F., V. E. Batista, F. R. Verri, H. M. Honório, C. C. de Mello, D. A. Almeida, and E. P. Pellizzer. “Platform-Switching Implants and Bone Preservation: A Systematic Review and Meta-Analysis.” *Int J Oral Maxillofac Surg* 45, no. 3 (March 2016): 332–45. <https://doi.org/10.1016/j.ijom.2015.11.009>.

Sanz-Martín, I., I. Sanz-Sánchez, A. Carrillo de Albornoz, E. Figuero, and M. Sanz. “Effects of Modified Abutment Characteristics on Peri-Implant Soft Tissue Health: A Systematic Review and Meta-Analysis.” *Clin Oral Implants Res* 29, no. 1 (January 2018): 118–29. <https://doi.org/10.1111/clr.13097>.

Sanz-Sánchez, I., I. Sanz-Martín, A. Carrillo de Albornoz, E. Figuero, and M. Sanz. “Biological Effect of the Abutment Material on the Stability of Peri-Implant Marginal Bone Levels: A Systematic Review and Meta-Analysis.” *Clin Oral Implants Res* 29 Suppl 18 (October 2018): 124–44. <https://doi.org/10.1111/clr.13293>.

Schiegnitz, E., and B. Al-Nawas. “Narrow-Diameter Implants: A Systematic Review and Meta-Analysis.” *Clin Oral Implants Res* 29 Suppl 16 (October 2018): 21–40. <https://doi.org/10.1111/clr.13272>.

Singh Gill, A., H. Morrissey, and A. Rahman. “A Systematic Review and Meta-Analysis Evaluating Antibiotic Prophylaxis in Dental Implants and Extraction Procedures.” *Medicina (Kaunas)* 54, no. 6 (December 1, 2018). <https://doi.org/10.3390/medicina54060095>.

Starch-Jensen, T., H. Aludden, M. Hallman, C. Dahlin, A. E. Christensen, and A. Mordenfeld. “A Systematic Review and Meta-Analysis of Long-Term Studies (Five or More Years) Assessing Maxillary Sinus Floor Augmentation.” *Int J Oral Maxillofac Surg* 47, no. 1 (January 2018): 103–16. <https://doi.org/10.1016/j.ijom.2017.05.001>.

Tavelli, L., S. Barootchi, T. V. N. Nguyen, M. Tattan, A. Ravidà, and H. L. Wang. “Efficacy of Tunnel Technique in the Treatment of Localized and Multiple Gingival Recessions: A Systematic Review and Meta-Analysis.” *J Periodontol* 89, no. 9 (September 2018): 1075–90. <https://doi.org/10.1002/jper.18-0066>.

Tavelli, L., A. Ravidà, G. H. Lin, F. S. Del Amo, M. Tattan, and H. L. Wang. “Comparison between Subepithelial Connective Tissue Graft and De-Epithelialized Gingival Graft: A Systematic Review and a Meta-Analysis.” *J Int Acad Periodontol* 21, no. 2 (April 1, 2019): 82–96.

Thoma, D. S., S. P. Bienz, E. Figuero, R. E. Jung, and I. Sanz-Martín. “Efficacy of Lateral Bone Augmentation Performed Simultaneously with Dental Implant Placement: A Systematic Review and Meta-Analysis.” *J Clin Periodontol* 46 Suppl 21 (June 2019): 257–76. <https://doi.org/10.1111/jcpe.13050>.

Tolentino da Rosa de Souza, P., M. Binhame Albini Martini, and L. Reis Azevedo-Alanis. “Do Short Implants Have Similar Survival Rates Compared to Standard Implants in Posterior Single Crown?: A Systematic Review and Meta-Analysis.” *Clin Implant Dent Relat Res* 20, no. 5 (October 2018): 890–901. <https://doi.org/10.1111/cid.12634>.

Tomasi, C., E. Regidor, A. Ortiz-Vigón, and J. Derks. “Efficacy of Reconstructive Surgical Therapy at Peri-Implantitis-Related Bone Defects. A Systematic Review and Meta-Analysis.” *J Clin Periodontol* 46 Suppl 21 (June 2019): 340–56. <https://doi.org/10.1111/jcpe.13070>.

Toti, P., S. Marchionni, G. B. Menchini-Fabris, S. Marconcini, U. Covani, and A. Barone. “Surgical Techniques Used in the Rehabilitation of Partially Edentulous Patients with Atrophic Posterior Mandibles: A Systematic Review and Meta-Analysis of Randomized Controlled Clinical Trials.” *J Craniomaxillofac Surg* 45, no. 8 (August 2017): 1236–45. <https://doi.org/10.1016/j.jcms.2017.04.011>.

Troiano, G., L. Laino, M. Dioguardi, G. Giannatempo, L. Lo Muzio, and L. Lo Russo. “Mandibular Class II Furcation Defect Treatment: Effects of the Addition of Platelet Concentrates to Open Flap: A Systematic Review and Meta-Analysis of Randomized Clinical Trials.” *J Periodontol* 87, no. 9 (September 2016): 1030–38. <https://doi.org/10.1902/jop.2016.160058>.

Troiano, G., L. Laino, K. Zhurakivska, M. Cicciù, L. Lo Muzio, and L. Lo Russo. “Addition of Enamel Matrix Derivatives to Bone Substitutes for the Treatment of Intrabony Defects: A Systematic Review, Meta-Analysis and Trial Sequential Analysis.” *J Clin Periodontol* 44, no. 7 (July 2017): 729–38. <https://doi.org/10.1111/jcpe.12742>.

Urban, I. A., E. Montero, A. Monje, and I. Sanz-Sánchez. “Effectiveness of Vertical Ridge Augmentation Interventions: A Systematic Review and Meta-Analysis.” *J Clin Periodontol* 46 Suppl 21 (June 2019): 319–39. <https://doi.org/10.1111/jcpe.13061>.

Wu, J., B. Li, and X. Lin. “Histological Outcomes of Sinus Augmentation for Dental Implants with Calcium Phosphate or Deproteinized Bovine Bone: A Systematic Review and Meta-Analysis.” *Int J Oral Maxillofac Surg* 45, no. 11 (November 2016): 1471–77. <https://doi.org/10.1016/j.ijom.2016.04.020>.

Yan, J., J. Zhang, Q. Zhang, X. Zhang, and K. Ji. “Effectiveness of Laser Adjunctive Therapy for Surgical Treatment of Gingival Recession with Flap Graft Techniques: A Systematic Review and Meta-Analysis.” *Lasers Med Sci* 33, no. 4 (May 2018): 899–908. <https://doi.org/10.1007/s10103-018-2440-x>.

Yan, M., M. Liu, M. Wang, F. Yin, and H. Xia. “The Effects of Er:YAG on the Treatment of Peri-Implantitis: A Meta-Analysis of Randomized Controlled Trials.” *Lasers Med Sci* 30, no. 7 (September 2015): 1843–53. <https://doi.org/10.1007/s10103-014-1692-3>.

Yang, J., Z. Cheng, and B. Shi. “Augmentation of the Alveolar Ridge Compared with Shorter Implants in Atrophic Jaws: A Meta-Analysis Based on Randomised Controlled Trials.” *Br J Oral Maxillofac Surg* 54, no. 1 (January 2016): 68–73. <https://doi.org/10.1016/j.bjoms.2015.10.011>.

Ye, P., T. Wei, Y. Wang, and Y. J. Cai. “Autologous Platelet Concentrates as Clinical Substitutes for Connective Tissue Graft in the Treatment of Miller Class I and II Gingival Recessions: An Updated Meta-Analysis.” *Int J Periodontics Restorative Dent* 40, no. 2 (April 2020): e53–63. <https://doi.org/10.11607/prd.4416>.

Yun, K. I., H. Choi, R. F. Wright, H. S. Ahn, B. M. Chang, and H. J. Kim. “Efficacy of Alveolar Vertical Distraction Osteogenesis and Autogenous Bone Grafting for Dental Implants: Systematic Review and Meta-Analysis.” *Int J Oral Maxillofac Implants* 31, no. 1 (February 2016): 26–36. <https://doi.org/10.11607/jomi.4479>.

Zhang, Z., Y. Zheng, and X. Bian. “Clinical Effect of Azithromycin as an Adjunct to Non-Surgical Treatment of Chronic Periodontitis: A Meta-Analysis of Randomized Controlled Clinical Trials.” *J Periodontal Res* 51, no. 3 (June 2016): 275–83. <https://doi.org/10.1111/jre.12319>.

Zhou, S., C. Sun, S. Huang, X. Wu, Y. Zhao, C. Pan, H. Wang, J. Liu, Q. Li, and Y. Kou. “Efficacy of Adjunctive Bioactive Materials in the Treatment of Periodontal Intrabony Defects: A Systematic Review and Meta-Analysis.” *Biomed Res Int* 2018 (2018): 8670832. <https://doi.org/10.1155/2018/8670832>.

## List S4. Excluded SRs after assessment of the title and abstract

**Primary reason for exclusion: did not fit the thematic area:**

Abduljabbar, T., F. Javed, A. Shah, M. S. Samer, F. Vohra, and Z. Akram. “Role of Lasers as an Adjunct to Scaling and Root Planing in Patients with Type 2 Diabetes Mellitus: A Systematic Review.” *Lasers Med Sci* 32, no. 2 (February 2017): 449–59. <https://doi.org/10.1007/s10103-016-2086-5>.

Abduljabbar, T., F. Vohra, F. Javed, and Z. Akram. “Antimicrobial Photodynamic Therapy Adjuvant to Non-Surgical Periodontal Therapy in Patients with Diabetes Mellitus: A Meta-Analysis.” *Photodiagnosis Photodyn Ther* 17 (March 2017): 138–46. <https://doi.org/10.1016/j.pdpdt.2016.11.008>.

Ahmed Elawady, D. M., A. F. Kaddah, and M. Talaat Khalifa. “Single vs 2 Implants on Peri-Implant Marginal Bone Level and Implant Failures in Mandibular Implant Overdentures: A Systematic Review With Meta-Analysis.” *J Evid Based Dent Pract* 17, no. 3 (September 2017): 216–25. <https://doi.org/10.1016/j.jebdp.2017.02.002>.

Akram, Z., T. Abduljabbar, S. Sauro, and U. Daood. “Effect of Photodynamic Therapy and Laser Alone as Adjunct to Scaling and Root Planing on Gingival Crevicular Fluid Inflammatory Proteins in Periodontal Disease: A Systematic Review.” *Photodiagnosis Photodyn Ther* 16 (December 2016): 142–53. <https://doi.org/10.1016/j.pdpdt.2016.09.004>.

Ali, Z., S. R. Baker, S. Shahrbaf, N. Martin, and M. V. Vettore. “Oral Health-Related Quality of Life after Prosthodontic Treatment for Patients with Partial Edentulism: A Systematic Review and Meta-Analysis.” *J Prosthet Dent* 121, no. 1 (January 2019): 59-68.e3. <https://doi.org/10.1016/j.prosdent.2018.03.003>.

Alqutaibi, A. Y. “Limited Evidence Suggests That the Immediate Placement of Dental Implants Into Infected Sites Versus Noninfected Sites in the Esthetic Zone Show Comparable Clinical Results.” *J Evid Based Dent Pract* 19, no. 2 (June 2019): 180–82. <https://doi.org/10.1016/j.jebdp.2019.05.002>.

Araujo, R. Z., J. F. Santiago Júnior, C. L. Cardoso, A. F. Benites Condezo, R. Moreira Júnior, and M. M. Curi. “Clinical Outcomes of Pterygoid Implants: Systematic Review and Meta-Analysis.” *J Craniomaxillofac Surg* 47, no. 4 (April 2019): 651–60. <https://doi.org/10.1016/j.jcms.2019.01.030>.

Ata-Ali, J., F. Ata-Ali, D. Peñarrocha-Oltra, and P. Galindo-Moreno. “What Is the Impact of Bisphosphonate Therapy upon Dental Implant Survival? A Systematic Review and Meta-Analysis.” *Clin Oral Implants Res* 27, no. 2 (February 2016): e38-46. <https://doi.org/10.1111/clr.12526>.

Atieh, M. A., W. J. Duncan, and C. M. Faggion. “Quality Assessment of Systematic Reviews on Oral Implants Placed Immediately into Fresh Extraction Sockets.” *Int J Oral Maxillofac Implants* 31, no. 2 (April 2016): 338–51. <https://doi.org/10.11607/jomi.4109>.

Awad, M. E., A. Altman, R. Elrefai, P. Shipman, S. Looney, and M. Elsalanty. “The Use of Vascularized Fibula Flap in Mandibular Reconstruction; A Comprehensive Systematic Review and Meta-Analysis of the Observational Studies.” *J Craniomaxillofac Surg* 47, no. 4 (April 2019): 629–41. <https://doi.org/10.1016/j.jcms.2019.01.037>.

Azaripour, A., S. Dittrich, C. J. F. Van Noorden, and B. Willershausen. “Efficacy of Photodynamic Therapy as Adjunct Treatment of Chronic Periodontitis: A Systematic Review and Meta-Analysis.” *Lasers Med Sci* 33, no. 2 (February 2018): 407–23. <https://doi.org/10.1007/s10103-017-2383-7>.

Bassir, S. H., K. El Kholy, C. Y. Chen, K. H. Lee, and G. Intini. “Outcome of Early Dental Implant Placement versus Other Dental Implant Placement Protocols: A Systematic Review and Meta-Analysis.” *J Periodontol* 90, no. 5 (May 2019): 493–506. <https://doi.org/10.1002/jper.18-0338>.

Bover-Ramos, F., J. Viña-Almunia, J. Cervera-Ballester, M. Peñarrocha-Diago, and B. García-Mira. “Accuracy of Implant Placement with Computer-Guided Surgery: A Systematic Review and Meta-Analysis Comparing Cadaver, Clinical, and In Vitro Studies.” *Int J Oral Maxillofac Implants* 33, no. 1 (February 2018): 101–15. <https://doi.org/10.11607/jomi.5556>.

Canellas, Jvds, P. J. D. Medeiros, Cmds Figueredo, R. G. Fischer, and F. G. Ritto. “Which Is the Best Choice after Tooth Extraction, Immediate Implant Placement or Delayed Placement with Alveolar Ridge Preservation? A Systematic Review and Meta-Analysis.” *J Craniomaxillofac Surg* 47, no. 11 (November 2019): 1793–1802. <https://doi.org/10.1016/j.jcms.2019.08.004>.

Carbajal Mejía, J. B., K. Wakabayashi, T. Nakano, and H. Yatani. “Marginal Bone Loss Around Dental Implants Inserted with Static Computer Assistance in Healed Sites: A Systematic Review and Meta-Analysis.” *Int J Oral Maxillofac Implants* 31, no. 4 (August 2016): 761–75. <https://doi.org/10.11607/jomi.4727>.

Chappuis, V., G. Avila-Ortiz, M. G. Araújo, and A. Monje. “Medication-Related Dental Implant Failure: Systematic Review and Meta-Analysis.” *Clin Oral Implants Res* 29 Suppl 16 (October 2018): 55–68. <https://doi.org/10.1111/clr.13137>.

Chen, H., G. Zhang, P. Weigl, and X. Gu. “Immediate Placement of Dental Implants into Infected versus Noninfected Sites in the Esthetic Zone: A Systematic Review and Meta-Analysis.” *J Prosthet Dent* 120, no. 5 (November 2018): 658–67. <https://doi.org/10.1016/j.prosdent.2017.12.008>.

Chen, J., M. Cai, J. Yang, T. Aldhohrah, and Y. Wang. “Immediate versus Early or Conventional Loading Dental Implants with Fixed Prostheses: A Systematic Review and Meta-Analysis of Randomized Controlled Clinical Trials.” *J Prosthet Dent* 122, no. 6 (December 2019): 516–36. <https://doi.org/10.1016/j.prosdent.2019.05.013>.

Chen, S., Q. Ou, X. Lin, and Y. Wang. “Comparison Between a Computer-Aided Surgical Template and the Free-Hand Method: A Systematic Review and Meta-Analysis.” *Implant Dent* 28, no. 6 (December 2019): 578–89. <https://doi.org/10.1097/id.0000000000000915>.

Chrcanovic, B. R., T. Albrektsson, and A. Wennerberg. “Bone Quality and Quantity and Dental Implant Failure: A Systematic Review and Meta-Analysis.” *Int J Prosthodont* 30, no. 3 (June 2017): 219–37. <https://doi.org/10.11607/ijp.5142>.

Cieplik, F., W. Buchalla, E. Hellwig, A. Al-Ahmad, K. A. Hiller, T. Maisch, and L. Karygianni. “Antimicrobial Photodynamic Therapy as an Adjunct for Treatment of Deep Carious Lesions-A Systematic Review.” *Photodiagnosis Photodyn Ther* 18 (June 2017): 54–62. <https://doi.org/10.1016/j.pdpdt.2017.01.005>.

Coulthard, P., M. Esposito, H. V. Worthington, and A. Jokstad. “WITHDRAWN: Interventions for Replacing Missing Teeth: Preprosthetic Surgery versus Dental Implants.” *Cochrane Database Syst Rev*, no. 12 (December 7, 2015): Cd003604. <https://doi.org/10.1002/14651858.CD003604.pub2>.

Cunha, A. C., A. M. A. da Veiga, D. Masterson, C. T. Mattos, L. I. Nojima, M. C. G. Nojima, and L. C. Maia. “How Do Geometry-Related Parameters Influence the Clinical Performance of Orthodontic Mini-Implants? A Systematic Review and Meta-Analysis.” *Int J Oral Maxillofac Surg* 46, no. 12 (December 2017): 1539–51. <https://doi.org/10.1016/j.ijom.2017.06.010>.

Daudt Polido, W., T. Aghaloo, T. W. Emmett, T. D. Taylor, and D. Morton. “Number of Implants Placed for Complete-Arch Fixed Prostheses: A Systematic Review and Meta-Analysis.” *Clin Oral Implants Res* 29 Suppl 16 (October 2018): 154–83. <https://doi.org/10.1111/clr.13312>.

Davoudi, A., R. Mosharraf, A. Akhavan, F. Zarei, S. Pourarz, and S. Iravani. “Effect of Laser Irradiation on Push-out Bond Strength of Dental Fiber Posts to Composite Resin Core Buildups: A Systematic Review and Meta-Analysis.” *Photodiagnosis Photodyn Ther* 27 (September 2019): 184–92. <https://doi.org/10.1016/j.pdpdt.2019.05.044>.

Day, P. F., M. Duggal, and H. Nazzal. “Interventions for Treating Traumatised Permanent Front Teeth: Avulsed (Knocked out) and Replanted.” *Cochrane Database Syst Rev* 2, no. 2 (February 5, 2019): Cd006542. <https://doi.org/10.1002/14651858.CD006542.pub3>.

Luna Gomes, J. M. de, C. A. A. Lemos, J. F. Santiago Junior, S. L. D. de Moraes, M. C. Goiato, and E. P. Pellizzer. “Optimal Number of Implants for Complete-Arch Implant-Supported Prostheses with a Follow-up of at Least 5 Years: A Systematic Review and Meta-Analysis.” *J Prosthet Dent* 121, no. 5 (May 2019): 766-774.e3. <https://doi.org/10.1016/j.prosdent.2018.06.001>.

Oliveira-Neto, O. B. de, F. T. Barbosa, C. F. de Sousa-Rodrigues, and F. J. C. de Lima. “Risk of Bias Assessment of Systematic Reviews Regarding Dental Implant Placement in Smokers: An Umbrella Systematic Review.” *J Prosthet Dent* 120, no. 2 (August 2018): 198–203. <https://doi.org/10.1016/j.prosdent.2017.12.026>.

Oliveira-Neto, O. B. de, C. A. Lemos, F. T. Barbosa, C. F. de Sousa-Rodrigues, and F. J. Camello de Lima. “Immediate Dental Implants Placed into Infected Sites Present a Higher Risk of Failure than Immediate Dental Implants Placed into Non-Infected Sites: Systematic Review and Meta-Analysis.” *Med Oral Patol Oral Cir Bucal* 24, no. 4 (July 1, 2019): e518–28. <https://doi.org/10.4317/medoral.22954>.

Souza Batista, V. E. de, A. J. Vechiato-Filho, J. F. Santiago, M. V. Sonego, F. R. Verri, D. M. Dos Santos, M. C. Goiato, and E. P. Pellizzer. “Clinical Viability of Single Implant-Retained Mandibular Overdentures: A Systematic Review and Meta-Analysis.” *Int J Oral Maxillofac Surg* 47, no. 9 (September 2018): 1166–77. <https://doi.org/10.1016/j.ijom.2018.01.021>.

Souza Batista, V. E. de, F. R. Verri, C. A. A. Lemos, R. S. Cruz, H. F. F. Oliveira, J. M. L. Gomes, and E. P. Pellizzer. “Should the Restoration of Adjacent Implants Be Splinted or Nonsplinted? A Systematic Review and Meta-Analysis.” *J Prosthet Dent* 121, no. 1 (January 2019): 41–51. <https://doi.org/10.1016/j.prosdent.2018.03.004>.

Del Fabbro, M., S. Corbella, P. Sequeira-Byron, I. Tsesis, E. Rosen, A. Lolato, and S. Taschieri. “Endodontic Procedures for Retreatment of Periapical Lesions.” *Cochrane Database Syst Rev* 10, no. 10 (October 19, 2016): Cd005511. <https://doi.org/10.1002/14651858.CD005511.pub3>.

Di Francesco, F., G. De Marco, U. A. Gironi Carnevale, M. Lanza, and A. Lanza. “The Number of Implants Required to Support a Maxillary Overdenture: A Systematic Review and Meta-Analysis.” *J Prosthodont Res* 63, no. 1 (January 2019): 15–24. <https://doi.org/10.1016/j.jpor.2018.08.006>.

Emery, R. W., S. A. Merritt, K. Lank, and J. D. Gibbs. “Accuracy of Dynamic Navigation for Dental Implant Placement-Model-Based Evaluation.” *J Oral Implantol* 42, no. 5 (October 2016): 399–405. <https://doi.org/10.1563/aaid-joi-D-16-00025>.

Escribano, M., E. Figuero, C. Martín, A. Tobías, J. Serrano, S. Roldán, and D. Herrera. “Efficacy of Adjunctive Anti-Plaque Chemical Agents: A Systematic Review and Network Meta-Analyses of the Turesky Modification of the Quigley and Hein Plaque Index.” *J Clin Periodontol* 43, no. 12 (December 2016): 1059–73. <https://doi.org/10.1111/jcpe.12616>.

Esposito, M., M. G. Grusovin, Y. S. Chew, P. Coulthard, and H. V. Worthington. “WITHDRAWN: Interventions for Replacing Missing Teeth: 1- versus 2-Stage Implant Placement.” *Cochrane Database Syst Rev* 5, no. 5 (May 23, 2018): Cd006698. <https://doi.org/10.1002/14651858.CD006698.pub3>.

Flügge, T., W. J. van der Meer, B. G. Gonzalez, K. Vach, D. Wismeijer, and P. Wang. “The Accuracy of Different Dental Impression Techniques for Implant-Supported Dental Prostheses: A Systematic Review and Meta-Analysis.” *Clin Oral Implants Res* 29 Suppl 16 (October 2018): 374–92. <https://doi.org/10.1111/clr.13273>.

Freitas da Silva, E. V., D. M. Dos Santos, M. V. Sonego, J. M. de Luna Gomes, E. P. Pellizzer, and M. C. Goiato. “Does the Presence of a Cantilever Influence the Survival and Success of Partial Implant-Supported Dental Prostheses? Systematic Review and Meta-Analysis.” *Int J Oral Maxillofac Implants* 33, no. 4 (August 2018): 815–23. <https://doi.org/10.11607/jomi.6413>.

Gomes, G. H., M. Y. O. Misawa, C. Fernandes, C. M. Pannuti, L. Saraiva, G. Huynh-Ba, and C. C. Villar. “A Systematic Review and Meta-Analysis of the Survival Rate of Implants Placed in Previously Failed Sites.” *Braz Oral Res* 32 (2018): e27. <https://doi.org/10.1590/1807-3107bor-2018.vol32.0027>.

Heitz-Mayfield, L. J., M. Aaboe, M. Araujo, J. B. Carrión, R. Cavalcanti, N. Cionca, D. Cochran, et al. “Group 4 ITI Consensus Report: Risks and Biologic Complications Associated with Implant Dentistry.” *Clin Oral Implants Res* 29 Suppl 16 (October 2018): 351–58. <https://doi.org/10.1111/clr.13307>.

Helmy, M. H. E., A. Y. Alqutaibi, A. A. El-Ella, and A. F. Shawky. “Effect of Implant Loading Protocols on Failure and Marginal Bone Loss with Unsplinted Two-Implant-Supported Mandibular Overdentures: Systematic Review and Meta-Analysis.” *Int J Oral Maxillofac Surg* 47, no. 5 (May 2018): 642–50. <https://doi.org/10.1016/j.ijom.2017.10.018>.

Huang, R. L., E. Kobayashi, K. Liu, and Q. Li. “Bone Graft Prefabrication Following the In Vivo Bioreactor Principle.” *EBioMedicine* 12 (October 2016): 43–54. <https://doi.org/10.1016/j.ebiom.2016.09.016>.

James, P., H. V. Worthington, C. Parnell, M. Harding, T. Lamont, A. Cheung, H. Whelton, and P. Riley. “Chlorhexidine Mouthrinse as an Adjunctive Treatment for Gingival Health.” *Cochrane Database Syst Rev* 3, no. 3 (March 31, 2017): Cd008676. <https://doi.org/10.1002/14651858.CD008676.pub2>.

Joda, T., W. Derksen, J. G. Wittneben, and S. Kuehl. “Static Computer-Aided Implant Surgery (s-CAIS) Analysing Patient-Reported Outcome Measures (PROMs), Economics and Surgical Complications: A Systematic Review.” *Clin Oral Implants Res* 29 Suppl 16 (October 2018): 359–73. <https://doi.org/10.1111/clr.13136>.

Kellesarian, S. V., V. R. Malignaggi, T. Abduljabbar, F. Vohra, H. Malmstrom, G. E. Romanos, and F. Javed. “Efficacy of Scaling and Root Planing with and without Adjunct Antimicrobial Photodynamic Therapy on the Expression of Cytokines in the Gingival Crevicular Fluid of Patients with Periodontitis: A Systematic Review.” *Photodiagnosis Photodyn Ther* 16 (December 2016): 76–84. <https://doi.org/10.1016/j.pdpdt.2016.08.009>.

Kellesarian, S. V., V. R. Malignaggi, H. A. Majoka, A. A. Al-Kheraif, T. V. Kellesarian, G. E. Romanos, and F. Javed. “Effect of Laser-Assisted Scaling and Root Planing on the Expression of pro-Inflammatory Cytokines in the Gingival Crevicular Fluid of Patients with Chronic Periodontitis: A Systematic Review.” *Photodiagnosis Photodyn Ther* 18 (June 2017): 63–77. <https://doi.org/10.1016/j.pdpdt.2017.02.010>.

Kellesarian, S. V., F. Qayyum, P. C. de Freitas, Z. Akram, and F. Javed. “Is Antimicrobial Photodynamic Therapy a Useful Therapeutic Protocol for Oral Decontamination? A Systematic Review and Meta-Analysis.” *Photodiagnosis Photodyn Ther* 20 (December 2017): 55–61. <https://doi.org/10.1016/j.pdpdt.2017.08.012>.

Ketabi, M., D. Deporter, and E. G. Atenafu. “A Systematic Review of Outcomes Following Immediate Molar Implant Placement Based on Recently Published Studies.” *Clin Implant Dent Relat Res* 18, no. 6 (December 2016): 1084–94. <https://doi.org/10.1111/cid.12390>.

Kim, J. S., S. M. Choi, J. H. Yoon, E. J. Lee, J. Yoon, S. H. Kwon, C. D. Yeo, et al. “What Affects Postoperative Sinusitis and Implant Failure after Dental Implant: A Meta-Analysis.” *Otolaryngol Head Neck Surg* 160, no. 6 (June 2019): 974–84. <https://doi.org/10.1177/0194599819829747>.

Kinaia, B. M., F. Ambrosio, M. Lamble, K. Hope, M. Shah, and A. L. Neely. “Soft Tissue Changes Around Immediately Placed Implants: A Systematic Review and Meta-Analyses With at Least 12 Months of Follow-Up After Functional Loading.” *J Periodontol* 88, no. 9 (September 2017): 876–86. <https://doi.org/10.1902/jop.2017.160698>.

Kumbargere Nagraj, S., P. Eachempati, E. Uma, V. P. Singh, N. M. Ismail, and E. Varghese. “Interventions for Managing Halitosis.” *Cochrane Database Syst Rev* 12, no. 12 (December 11, 2019): Cd012213. <https://doi.org/10.1002/14651858.CD012213.pub2>.

Lemos, C. A., V. E. de Souza Batista, D. A. Almeida, J. F. Santiago Júnior, F. R. Verri, and E. P. Pellizzer. “Evaluation of Cement-Retained versus Screw-Retained Implant-Supported Restorations for Marginal Bone Loss: A Systematic Review and Meta-Analysis.” *J Prosthet Dent* 115, no. 4 (April 2016): 419–27. <https://doi.org/10.1016/j.prosdent.2015.08.026>.

Li, Z. B., and K. Li. “Prophylactic Antibiotics Can Prevent Early Implant Failure, but Postoperative Antibiotics May Not Be Beneficial for Dental Implant Placement.” *J Evid Based Dent Pract* 19, no. 4 (December 2019): 101339. <https://doi.org/10.1016/j.jebdp.2019.101339>.

Lian, M., K. Zhao, Y. Feng, and Q. Yao. “Prognosis of Combining Remaining Teeth and Implants in Double-Crown-Retained Removable Dental Prostheses: A Systematic Review and Meta-Analysis.” *Int J Oral Maxillofac Implants* 33, no. 2 (April 2018): 281–97. <https://doi.org/10.11607/jomi.5796>.

Lim, G., G. H. Lin, A. Monje, H. L. Chan, and H. L. Wang. “Wound Healing Complications Following Guided Bone Regeneration for Ridge Augmentation: A Systematic Review and Meta-Analysis.” *Int J Oral Maxillofac Implants* 33, no. 1 (February 2018): 41–50. <https://doi.org/10.11607/jomi.5581>.

Liu, W., Y. Cao, L. Dong, Y. Zhu, Y. Wu, Z. Lv, Z. Iheozor-Ejiofor, and C. Li. “Periodontal Therapy for Primary or Secondary Prevention of Cardiovascular Disease in People with Periodontitis.” *Cochrane Database Syst Rev* 12, no. 12 (December 31, 2019): Cd009197. <https://doi.org/10.1002/14651858.CD009197.pub4>.

Lund, B., M. Hultin, S. Tranaeus, A. Naimi-Akbar, and B. Klinge. “Complex Systematic Review - Perioperative Antibiotics in Conjunction with Dental Implant Placement.” *Clin Oral Implants Res* 26 Suppl 11 (September 2015): 1–14. <https://doi.org/10.1111/clr.12637>.

Manfredi, M., L. Figini, M. Gagliani, and G. Lodi. “Single versus Multiple Visits for Endodontic Treatment of Permanent Teeth.” *Cochrane Database Syst Rev* 12, no. 12 (December 1, 2016): Cd005296. <https://doi.org/10.1002/14651858.CD005296.pub3>.

Manresa, C., E. C. Sanz-Miralles, J. Twigg, and M. Bravo. “Supportive Periodontal Therapy (SPT) for Maintaining the Dentition in Adults Treated for Periodontitis.” *Cochrane Database Syst Rev* 1, no. 1 (January 1, 2018): Cd009376. <https://doi.org/10.1002/14651858.CD009376.pub2>.

Marcello-Machado, R. M., F. Faot, A. J. Schuster, G. G. Nascimento, and A. A. Del Bel Cury. “Mini-Implants and Narrow Diameter Implants as Mandibular Overdenture Retainers: A Systematic Review and Meta-Analysis of Clinical and Radiographic Outcomes.” *J Oral Rehabil* 45, no. 2 (February 2018): 161–83. <https://doi.org/10.1111/joor.12585>.

Mathur, A., D. Gopalakrishnan, V. Mehta, S. A. Rizwan, S. H. Shetiya, and S. Bagwe. “Efficacy of Green Tea-Based Mouthwashes on Dental Plaque and Gingival Inflammation: A Systematic Review and Meta-Analysis.” *Indian J Dent Res* 29, no. 2 (April 2018): 225–32. <https://doi.org/10.4103/ijdr.IJDR_493_17>.

Mello, C. C., C. A. A. Lemos, F. R. Verri, D. M. Dos Santos, M. C. Goiato, and E. P. Pellizzer. “Immediate Implant Placement into Fresh Extraction Sockets versus Delayed Implants into Healed Sockets: A Systematic Review and Meta-Analysis.” *Int J Oral Maxillofac Surg* 46, no. 9 (September 2017): 1162–77. <https://doi.org/10.1016/j.ijom.2017.03.016>.

Meursinge Reynders, R., L. Ronchi, L. Ladu, N. Di Girolamo, J. de Lange, N. Roberts, and S. Mickan. “Barriers and Facilitators to the Implementation of Orthodontic Mini-Implants in Clinical Practice: A Protocol for a Systematic Review and Meta-Analysis.” *Syst Rev* 5 (February 5, 2016): 22. <https://doi.org/10.1186/s13643-016-0198-4>.

Moldovan, O., H. Rudolph, and R. G. Luthardt. “Biological Complications of Removable Dental Prostheses in the Moderately Reduced Dentition: A Systematic Literature Review.” *Clin Oral Investig* 22, no. 7 (September 2018): 2439–61. <https://doi.org/10.1007/s00784-018-2522-y>.

Monje, A., H. L. Chan, P. Galindo-Moreno, B. Elnayef, F. Suarez-Lopez del Amo, F. Wang, and H. L. Wang. “Alveolar Bone Architecture: A Systematic Review and Meta-Analysis.” *J Periodontol* 86, no. 11 (November 2015): 1231–48. <https://doi.org/10.1902/jop.2015.150263>.

Nibali, L., V. P. Koidou, T. Hamborg, and N. Donos. “Empirical or Microbiologically Guided Systemic Antimicrobials as Adjuncts to Non-Surgical Periodontal Therapy? A Systematic Review.” *J Clin Periodontol* 46, no. 10 (October 2019): 999–1012. <https://doi.org/10.1111/jcpe.13164>.

Padmanabhan, H., S. M. Kumar, and V. A. Kumar. “Single Implant Retained Overdenture Treatment Protocol: A Systematic Review and Meta-Analysis.” *J Prosthodont* 29, no. 4 (April 2020): 287–97. <https://doi.org/10.1111/jopr.13133>.

Park, J. H., J. Y. Lee, S. W. Shin, and H. J. Kim. “Effect of Conversion to Implant-Assisted Removable Partial Denture in Patients with Mandibular Kennedy Classification Ⅰ: A Systematic Review and Meta-Analysis.” *Clin Oral Implants Res* 31, no. 4 (April 2020): 360–73. <https://doi.org/10.1111/clr.13574>.

Parkin, N., P. E. Benson, B. Thind, A. Shah, I. Khalil, and S. Ghafoor. “Open versus Closed Surgical Exposure of Canine Teeth That Are Displaced in the Roof of the Mouth.” *Cochrane Database Syst Rev* 8, no. 8 (August 21, 2017): Cd006966. <https://doi.org/10.1002/14651858.CD006966.pub3>.

Pauletto, P., E. Ruales-Carrera, Tmsv Gonçalves, A. G. Philippi, N. Donos, and L. A. Mezzomo. “Fixed and Removable Full-Arch Restorations Supported by Short (≤ 8-Mm) Dental Implants In the Mandible: A Systematic Review and Meta-Analysis.” *Int J Oral Maxillofac Implants* 34, no. 4 (August 2019): 873–85. <https://doi.org/10.11607/jomi.7241>.

Permuy, M., M. López-Peña, A. González-Cantalapiedra, and F. Muñoz. “Melatonin: A Review of Its Potential Functions and Effects on Dental Diseases.” *Int J Mol Sci* 18, no. 4 (April 19, 2017). <https://doi.org/10.3390/ijms18040865>.

Pieralli, S., R. J. Kohal, K. Rabel, M. von Stein-Lausnitz, K. Vach, and B. C. Spies. “Clinical Outcomes of Partial and Full-Arch All-Ceramic Implant-Supported Fixed Dental Prostheses. A Systematic Review and Meta-Analysis.” *Clin Oral Implants Res* 29 Suppl 18 (October 2018): 224–36. <https://doi.org/10.1111/clr.13345>.

Pol, C. W. P., G. M. Raghoebar, W. Kerdijk, G. C. Boven, M. S. Cune, and H. J. A. Meijer. “A Systematic Review and Meta-Analysis of 3-Unit Fixed Dental Prostheses: Are the Results of 2 Abutment Implants Comparable to the Results of 2 Abutment Teeth?” *J Oral Rehabil* 45, no. 2 (February 2018): 147–60. <https://doi.org/10.1111/joor.12575>.

Qi, N., W. J. Li, and H. Tian. “A Systematic Review of Animal and Clinical Studies on the Use of Scaffolds for Urethral Repair.” *J Huazhong Univ Sci Technolog Med Sci* 36, no. 1 (February 2016): 111–17. <https://doi.org/10.1007/s11596-016-1551-5>.

Rabel, K., B. C. Spies, S. Pieralli, K. Vach, and R. J. Kohal. “The Clinical Performance of All-Ceramic Implant-Supported Single Crowns: A Systematic Review and Meta-Analysis.” *Clin Oral Implants Res* 29 Suppl 18 (October 2018): 196–223. <https://doi.org/10.1111/clr.13337>.

Raico Gallardo, Y. N., I. R. T. da Silva-Olivio, E. Mukai, S. Morimoto, N. Sesma, and L. Cordaro. “Accuracy Comparison of Guided Surgery for Dental Implants According to the Tissue of Support: A Systematic Review and Meta-Analysis.” *Clin Oral Implants Res* 28, no. 5 (May 2017): 602–12. <https://doi.org/10.1111/clr.12841>.

Ramamoorthi, M., A. Narvekar, and S. Esfandiari. “A Meta-Analysis of Retention Systems for Implant-Supported Prostheses in Partially Edentulous Jaws.” *J Prosthet Dent* 118, no. 5 (November 2017): 587–95. <https://doi.org/10.1016/j.prosdent.2016.11.019>.

Sivarajan, S., L. P. Ringgingon, M. M. S. Fayed, and M. C. Wey. “The Effect of Micro-Osteoperforations on the Rate of Orthodontic Tooth Movement: A Systematic Review and Meta-Analysis.” *Am J Orthod Dentofacial Orthop* 157, no. 3 (March 2020): 290–304. <https://doi.org/10.1016/j.ajodo.2019.10.009>.

Souza, E., A. C. Medeiros, B. C. Gurgel, and C. Sarmento. “Antimicrobial Photodynamic Therapy in the Treatment of Aggressive Periodontitis: A Systematic Review and Meta-Analysis.” *Lasers Med Sci* 31, no. 1 (January 2016): 187–96. <https://doi.org/10.1007/s10103-015-1836-0>.

Srinivasan, M., N. A. Makarov, F. R. Herrmann, and F. Müller. “Implant Survival in 1- versus 2-Implant Mandibular Overdentures: A Systematic Review and Meta-Analysis.” *Clin Oral Implants Res* 27, no. 1 (January 2016): 63–72. <https://doi.org/10.1111/clr.12513>.

Stavropoulos, A., K. Bertl, P. Pietschmann, N. Pandis, M. Schiødt, and B. Klinge. “The Effect of Antiresorptive Drugs on Implant Therapy: Systematic Review and Meta-Analysis.” *Clin Oral Implants Res* 29 Suppl 18 (October 2018): 54–92. <https://doi.org/10.1111/clr.13282>.

Stein-Lausnitz, M. von, H. J. Nickenig, S. Wolfart, K. Neumann, A. von Stein-Lausnitz, B. C. Spies, and F. Beuer. “Survival Rates and Complication Behaviour of Tooth Implant-Supported, Fixed Dental Prostheses: A Systematic Review and Meta-Analysis.” *J Dent* 88 (September 2019): 103167. <https://doi.org/10.1016/j.jdent.2019.07.005>.

Tahmaseb, A., V. Wu, D. Wismeijer, W. Coucke, and C. Evans. “The Accuracy of Static Computer-Aided Implant Surgery: A Systematic Review and Meta-Analysis.” *Clin Oral Implants Res* 29 Suppl 16 (October 2018): 416–35. <https://doi.org/10.1111/clr.13346>.

Teshome, A., and A. Yitayeh. “The Effect of Periodontal Therapy on Glycemic Control and Fasting Plasma Glucose Level in Type 2 Diabetic Patients: Systematic Review and Meta-Analysis.” *BMC Oral Health* 17, no. 1 (July 30, 2016): 31. <https://doi.org/10.1186/s12903-016-0249-1>.

Ting, M., M. S. Tenaglia, G. H. Jones, and J. B. Suzuki. “Surgical and Patient Factors Affecting Marginal Bone Levels Around Dental Implants: A Comprehensive Overview of Systematic Reviews.” *Implant Dent* 26, no. 2 (April 2017): 303–15. <https://doi.org/10.1097/id.0000000000000565>.

Valles, C., X. Rodríguez-Ciurana, M. Clementini, M. Baglivo, B. Paniagua, and J. Nart. “Influence of Subcrestal Implant Placement Compared with Equicrestal Position on the Peri-Implant Hard and Soft Tissues around Platform-Switched Implants: A Systematic Review and Meta-Analysis.” *Clin Oral Investig* 22, no. 2 (March 2018): 555–70. <https://doi.org/10.1007/s00784-017-2301-1>.

Van der Sluijs, M., E. Van der Sluijs, F. Van der Weijden, and D. E. Slot. “The Effect on Clinical Parameters of Periodontal Inflammation Following Non-Surgical Periodontal Therapy with Ultrasonics and Chemotherapeutic Cooling Solutions: A Systematic Review.” *J Clin Periodontol* 43, no. 12 (December 2016): 1074–85. <https://doi.org/10.1111/jcpe.12613>.

Van der Weijden, F. A., E. Van der Sluijs, S. G. Ciancio, and D. E. Slot. “Can Chemical Mouthwash Agents Achieve Plaque/Gingivitis Control?” *Dent Clin North Am* 59, no. 4 (October 2015): 799–829. <https://doi.org/10.1016/j.cden.2015.06.002>.

Villar, C. C., C. M. Pannuti, D. M. Nery, C. M. Morillo, M. J. Carmona, and G. A. Romito. “Effectiveness of Intraoral Chlorhexidine Protocols in the Prevention of Ventilator-Associated Pneumonia: Meta-Analysis and Systematic Review.” *Respir Care* 61, no. 9 (September 2016): 1245–59. <https://doi.org/10.4187/respcare.04610>.

Vohra, F., Z. Akram, S. H. Safii, R. D. Vaithilingam, A. Ghanem, K. Sergis, and F. Javed. “Role of Antimicrobial Photodynamic Therapy in the Treatment of Aggressive Periodontitis: A Systematic Review.” *Photodiagnosis Photodyn Ther* 13 (March 2016): 139–47. <https://doi.org/10.1016/j.pdpdt.2015.06.010>.

Werner, H., M. Hakeberg, L. Dahlström, M. Eriksson, P. Sjögren, A. Strandell, T. Svanberg, L. Svensson, and U. Wide Boman. “Psychological Interventions for Poor Oral Health: A Systematic Review.” *J Dent Res* 95, no. 5 (May 2016): 506–14. <https://doi.org/10.1177/0022034516628506>.

Xue, D., and Y. Zhao. “Clinical Effectiveness of Adjunctive Antimicrobial Photodynamic Therapy for Residual Pockets during Supportive Periodontal Therapy: A Systematic Review and Meta-Analysis.” *Photodiagnosis Photodyn Ther* 17 (March 2017): 127–33. <https://doi.org/10.1016/j.pdpdt.2016.11.011>.

Yi, J., M. Ge, M. Li, C. Li, Y. Li, X. Li, and Z. Zhao. “Comparison of the Success Rate between Self-Drilling and Self-Tapping Miniscrews: A Systematic Review and Meta-Analysis.” *Eur J Orthod* 39, no. 3 (June 1, 2017): 287–93. <https://doi.org/10.1093/ejo/cjw036>.

Zhang, J., N. Ab Malik, C. McGrath, and O. Lam. “The Effect of Antiseptic Oral Sprays on Dental Plaque and Gingival Inflammation: A Systematic Review and Meta-Analysis.” *Int J Dent Hyg* 17, no. 1 (February 2019): 16–26. <https://doi.org/10.1111/idh.12331>.

Zhang, J. N., H. P. Lu, X. C. Bao, Y. Shi, and M. H. Zhang. “Evaluation of the Long-Term Stability of Micro-Screws under Different Loading Protocols: A Systematic Review.” *Braz Oral Res* 33 (June 10, 2019): e046. <https://doi.org/10.1590/1807-3107bor-2019.vol33.0046>.

Zhao, K., F. Wang, W. Huang, and Y. Wu. “Clinical Outcomes of Vertical Distraction Osteogenesis for Dental Implantation: A Systematic Review and Meta-Analysis.” *Int J Oral Maxillofac Implants* 33, no. 3 (June 2018): 549–64. <https://doi.org/10.11607/jomi.6140>.

Zhou, W., Z. Liu, L. Song, C. L. Kuo, and D. M. Shafer. “Clinical Factors Affecting the Accuracy of Guided Implant Surgery-A Systematic Review and Meta-Analysis.” *J Evid Based Dent Pract* 18, no. 1 (March 2018): 28–40. <https://doi.org/10.1016/j.jebdp.2017.07.007>.

Zhou, X., J. Yang, L. Wu, X. Tang, Y. Mou, W. Sun, Q. Hu, and S. Xie. “Evaluation of the Effect of Implants Placed in Preserved Sockets Versus Fresh Sockets on Tissue Preservation and Esthetics: A Meta-Analysis and Systematic Review.” *J Evid Based Dent Pract* 19, no. 4 (December 2019): 101336. <https://doi.org/10.1016/j.jebdp.2019.05.015>.

Zhuang, J., D. Zhao, Y. Wu, and C. Xu. “Evaluation of Outcomes of Dental Implants Inserted by Flapless or Flapped Procedure: A Meta-Analysis.” *Implant Dent* 27, no. 5 (October 2018): 588–98. <https://doi.org/10.1097/id.0000000000000820>.

**Primary reason for exclusion: involved risk factors:**

Akram, Z., M. A. Raffat, S. Saad Shafqat, S. Mirza, and S. Ikram. “Clinical Efficacy of Photodynamic Therapy as an Adjunct to Scaling and Root Planing in the Treatment of Chronic Periodontitis among Cigarette Smokers: A Systematic Review and Meta-Analysis.” *Photodiagnosis Photodyn Ther* 26 (June 2019): 334–41. <https://doi.org/10.1016/j.pdpdt.2019.04.027>.

Al-Hamoudi, N. “Is Antimicrobial Photodynamic Therapy an Effective Treatment for Chronic Periodontitis in Diabetes Mellitus and Cigarette Smokers: A Systematic Review and Meta-Analysis.” *Photodiagnosis Photodyn Ther* 19 (September 2017): 375–82. <https://doi.org/10.1016/j.pdpdt.2017.05.018>.

Assem, N. Z., M. L. F. Alves, A. B. Lopes, E. C. Junior Gualberto, V. G. Garcia, and L. H. Theodoro. “Antibiotic Therapy as an Adjunct to Scaling and Root Planing in Smokers: A Systematic Review and Meta-Analysis.” *Braz Oral Res* 31 (July 3, 2017): e67. <https://doi.org/10.1590/1807-3107BOR-2017.vol31.0067>.

Cao, R., Q. Li, Q. Wu, M. Yao, Y. Chen, and H. Zhou. “Effect of Non-Surgical Periodontal Therapy on Glycemic Control of Type 2 Diabetes Mellitus: A Systematic Review and Bayesian Network Meta-Analysis.” *BMC Oral Health* 19, no. 1 (August 6, 2019): 176. <https://doi.org/10.1186/s12903-019-0829-y>.

Cardona, A., A. Balouch, M. M. Abdul, P. P. Sedghizadeh, and R. Enciso. “Efficacy of Chlorhexidine for the Prevention and Treatment of Oral Mucositis in Cancer Patients: A Systematic Review with Meta-Analyses.” *J Oral Pathol Med* 46, no. 9 (October 2017): 680–88. <https://doi.org/10.1111/jop.12549>.

Chrcanovic, B. R., T. Albrektsson, and A. Wennerberg. “Dental Implants in Irradiated versus Nonirradiated Patients: A Meta-Analysis.” *Head Neck* 38, no. 3 (March 2016): 448–81. <https://doi.org/10.1002/hed.23875>.

Chrcanovic, Bruno Ramos, Tomas Albrektsson, and Ann Wennerberg. “Dental Implants in Patients Receiving Chemotherapy: A Meta-Analysis.” *Implant Dentistry* 25, no. 2 (April 2016): 261–71. <https://doi.org/10.1097/ID.0000000000000388>.

Glurich, I., and A. Acharya. “Updates from the Evidence Base Examining Association between Periodontal Disease and Type 2 Diabetes Mellitus: Current Status and Clinical Relevance.” *Curr Diab Rep* 19, no. 11 (November 6, 2019): 121. <https://doi.org/10.1007/s11892-019-1228-0>.

Grellmann, A. P., C. S. Sfreddo, J. Maier, T. L. Lenzi, and F. B. Zanatta. “Systemic Antimicrobials Adjuvant to Periodontal Therapy in Diabetic Subjects: A Meta-Analysis.” *J Clin Periodontol* 43, no. 3 (March 2016): 250–60. <https://doi.org/10.1111/jcpe.12514>.

Grisa, A., and A. Veitz-Keenan. “Is Osteoporosis a Risk Factor for Implant Survival or Failure?” *Evid Based Dent* 19, no. 2 (June 2018): 51–52. <https://doi.org/10.1038/sj.ebd.6401307>.

Lin, C. Y., Z. Chen, W. L. Pan, and H. L. Wang. “Is History of Periodontal Disease Still a Negative Risk Indicator for Peri-Implant Health Under Supportive Post-Implant Treatment Coverage? A Systematic Review and Meta-Analysis.” *Int J Oral Maxillofac Implants* 35, no. 1 (February 2020): 52–62. <https://doi.org/10.11607/jomi.7714>.

Schimmel, M., M. Srinivasan, G. McKenna, and F. Müller. “Effect of Advanced Age and/or Systemic Medical Conditions on Dental Implant Survival: A Systematic Review and Meta-Analysis.” *Clin Oral Implants Res* 29 Suppl 16 (October 2018): 311–30. <https://doi.org/10.1111/clr.13288>.

Sendyk, D. I., E. S. Rovai, C. M. Pannuti, M. C. Deboni, W. R. Sendyk, and A. Wennerberg. “Dental Implant Loss in Older versus Younger Patients: A Systematic Review and Meta-Analysis of Prospective Studies.” *J Oral Rehabil* 44, no. 3 (March 2017): 229–36. <https://doi.org/10.1111/joor.12465>.

Simpson, T. C., J. C. Weldon, H. V. Worthington, I. Needleman, S. H. Wild, D. R. Moles, B. Stevenson, S. Furness, and Z. Iheozor-Ejiofor. “Treatment of Periodontal Disease for Glycaemic Control in People with Diabetes Mellitus.” *Cochrane Database Syst Rev* 2015, no. 11 (November 6, 2015): Cd004714. <https://doi.org/10.1002/14651858.CD004714.pub3>.

Smith Nobrega, A., J. F. Santiago, D. A. de Faria Almeida, D. M. Dos Santos, E. P. Pellizzer, and M. C. Goiato. “Irradiated Patients and Survival Rate of Dental Implants: A Systematic Review and Meta-Analysis.” *J Prosthet Dent* 116, no. 6 (December 2016): 858–66. <https://doi.org/10.1016/j.prosdent.2016.04.025>.

Souto, M. L. S., E. S. Rovai, J. A. Ganhito, M. Holzhausen, L. Chambrone, and C. M. Pannuti. “Efficacy of Systemic Antibiotics in Nonsurgical Periodontal Therapy for Diabetic Subjects: A Systematic Review and Meta-Analysis.” *Int Dent J* 68, no. 4 (August 2018): 207–20. <https://doi.org/10.1111/idj.12384>.

Srinivasan, M., S. Meyer, A. Mombelli, and F. Müller. “Dental Implants in the Elderly Population: A Systematic Review and Meta-Analysis.” *Clin Oral Implants Res* 28, no. 8 (August 2017): 920–30. <https://doi.org/10.1111/clr.12898>.

Theodoridis, C., A. Grigoriadis, G. Menexes, and I. Vouros. “Outcomes of Implant Therapy in Patients with a History of Aggressive Periodontitis. A Systematic Review and Meta-Analysis.” *Clin Oral Investig* 21, no. 2 (March 2017): 485–503. <https://doi.org/10.1007/s00784-016-2026-6>.

Yap, K. C. H., and S. J. Pulikkotil. “Systemic Doxycycline as an Adjunct to Scaling and Root Planing in Diabetic Patients with Periodontitis: A Systematic Review and Meta-Analysis.” *BMC Oral Health* 19, no. 1 (September 5, 2019): 209. <https://doi.org/10.1186/s12903-019-0873-7>.

**Primary reason for exclusion: not performed on humans:**

Abduljabbar, T., S. V. Kellesarian, F. Vohra, Z. Akram, G. A. Kotsakis, M. Yunker, G. E. Romanos, and F. Javed. “Effect of Growth Hormone Supplementation on Osseointegration: A Systematic Review and Meta-Analyses.” *Implant Dent* 26, no. 4 (August 2017): 613–20. <https://doi.org/10.1097/id.0000000000000616>.

Alberton Nuernberg, M. A., D. M. Janjacomo Miessi, C. A. Ivanaga, M. Bocalon Olivo, E. Ervolino, V. Gouveia Garcia, M. Wainwright, and L. H. Theodoro. “Influence of Antimicrobial Photodynamic Therapy as an Adjunctive to Scaling and Root Planing on Alveolar Bone Loss: A Systematic Review and Meta-Analysis of Animal Studies.” *Photodiagnosis Photodyn Ther* 25 (March 2019): 354–63. <https://doi.org/10.1016/j.pdpdt.2019.01.020>.

Alenezi, A., B. Chrcanovic, and A. Wennerberg. “Effects of Local Drug and Chemical Compound Delivery on Bone Regeneration Around Dental Implants in Animal Models: A Systematic Review and Meta-Analysis.” *Int J Oral Maxillofac Implants* 33, no. 1 (February 2018): e1–18. <https://doi.org/10.11607/jomi.6333>.

Basudan, A. M., M. Y. Shaheen, R. B. de Vries, Jjjp van den Beucken, J. A. Jansen, and H. S. Alghamdi. “Antiosteoporotic Drugs to Promote Bone Regeneration Related to Titanium Implants: A Systematic Review and Meta-Analysis.” *Tissue Eng Part B Rev* 25, no. 2 (April 2019): 89–99. <https://doi.org/10.1089/ten.TEB.2018.0120>.

Bertl, K., I. Steiner, N. Pandis, K. Buhlin, B. Klinge, and A. Stavropoulos. “Statins in Nonsurgical and Surgical Periodontal Therapy. A Systematic Review and Meta-Analysis of Preclinical in Vivo Trials.” *J Periodontal Res* 53, no. 3 (June 2018): 267–87. <https://doi.org/10.1111/jre.12514>.

Haag, P. A., V. Steiger-Ronay, and P. R. Schmidlin. “The in Vitro Antimicrobial Efficacy of PDT against Periodontopathogenic Bacteria.” *Int J Mol Sci* 16, no. 11 (November 13, 2015): 27327–38. <https://doi.org/10.3390/ijms161126027>.

Hindy, A., F. Farahmand, and F. S. Tabatabaei. “In Vitro Biological Outcome of Laser Application for Modification or Processing of Titanium Dental Implants.” *Lasers Med Sci* 32, no. 5 (July 2017): 1197–1206. <https://doi.org/10.1007/s10103-017-2217-7>.

Kellesarian, S. V., T. Abduljabbar, F. Vohra, V. R. Malignaggi, H. Malmstrom, G. E. Romanos, and F. Javed. “Role of Local Alendronate Delivery on the Osseointegration of Implants: A Systematic Review and Meta-Analysis.” *Int J Oral Maxillofac Surg* 46, no. 7 (July 2017): 912–21. <https://doi.org/10.1016/j.ijom.2017.03.009>.

Rakic, M., P. Galindo-Moreno, A. Monje, S. Radovanovic, H. L. Wang, D. Cochran, A. Sculean, and L. Canullo. “How Frequent Does Peri-Implantitis Occur? A Systematic Review and Meta-Analysis.” *Clin Oral Investig* 22, no. 4 (May 2018): 1805–16. <https://doi.org/10.1007/s00784-017-2276-y>.

Shanbhag, S., N. Pandis, K. Mustafa, J. R. Nyengaard, and A. Stavropoulos. “Bone Tissue Engineering in Oral Peri-Implant Defects in Preclinical in Vivo Research: A Systematic Review and Meta-Analysis.” *J Tissue Eng Regen Med* 12, no. 1 (January 2018): e336–49. <https://doi.org/10.1002/term.2412>.

Shi, J., Y. Li, Y. Gu, S. Qiao, X. Zhang, and H. Lai. “Effect of Titanium Implants with Strontium Incorporation on Bone Apposition in Animal Models: A Systematic Review and Meta-Analysis.” *Sci Rep* 7, no. 1 (November 14, 2017): 15563. <https://doi.org/10.1038/s41598-017-15488-1>.

Wang, B., J. Shao, J. Fu, J. A. Jansen, X. F. Walboomers, C. R. Hooijmans, J. Van Luijk, and F. Yang. “Topical Host-Modulating Therapy for Periodontal Regeneration: A Systematic Review and Meta-Analysis.” *Tissue Eng Part B Rev* 25, no. 6 (December 2019): 526–43. <https://doi.org/10.1089/ten.TEB.2019.0184>.

Yan, X. Z., F. Yang, J. A. Jansen, R. B. de Vries, and J. J. van den Beucken. “Cell-Based Approaches in Periodontal Regeneration: A Systematic Review and Meta-Analysis of Periodontal Defect Models in Animal Experimental Work.” *Tissue Eng Part B Rev* 21, no. 5 (October 2015): 411–26. <https://doi.org/10.1089/ten.TEB.2015.0049>.

**Primary reason for exclusion: no pair-wise meta-analysis:**

Al-Moraissi, E. A., N. H. Altairi, B. Abotaleb, G. Al-Iryani, E. Halboub, and M. S. Alakhali. “What Is the Most Effective Rehabilitation Method for Posterior Maxillas With 4 to 8 Mm of Residual Alveolar Bone Height Below the Maxillary Sinus With Implant-Supported Prostheses? A Frequentist Network Meta-Analysis.” *J Oral Maxillofac Surg* 77, no. 1 (January 2019): 70.e1-70.e33. <https://doi.org/10.1016/j.joms.2018.08.009>.

Barbato, L., F. Selvaggi, Z. Kalemaj, J. Buti, E. Bendinelli, M. Marca, and F. Cairo. “Clinical Efficacy of Minimally Invasive Surgical (MIS) and Non-Surgical (MINST) Treatments of Periodontal Intra-Bony Defect. A Systematic Review and Network Meta-Analysis of RCT’s.” *Clin Oral Investig* 24, no. 3 (March 2020): 1125–35. <https://doi.org/10.1007/s00784-020-03229-0>.

Cairo, F., U. Pagliaro, J. Buti, M. Baccini, F. Graziani, P. Tonelli, G. Pagavino, and M. S. Tonetti. “Root Coverage Procedures Improve Patient Aesthetics. A Systematic Review and Bayesian Network Meta-Analysis.” *J Clin Periodontol* 43, no. 11 (November 2016): 965–75. <https://doi.org/10.1111/jcpe.12603>.

Figuero, E., D. Herrera, A. Tobías, J. Serrano, S. Roldán, M. Escribano, and C. Martín. “Efficacy of Adjunctive Anti-Plaque Chemical Agents in Managing Gingivitis: A Systematic Review and Network Meta-Analyses.” *J Clin Periodontol* 46, no. 7 (July 2019): 723–39. <https://doi.org/10.1111/jcpe.13127>.

Iocca, O., A. Farcomeni, S. Pardiñas Lopez, and H. S. Talib. “Alveolar Ridge Preservation after Tooth Extraction: A Bayesian Network Meta-Analysis of Grafting Materials Efficacy on Prevention of Bone Height and Width Reduction.” *J Clin Periodontol* 44, no. 1 (January 2017): 104–14. <https://doi.org/10.1111/jcpe.12633>.

Jagannathan, N., A. Acharya, O. Yi Farn, K. Y. Li, L. Nibali, and G. Pelekos. “Disease Severity, Debridement Approach and Timing of Drug Modify Outcomes of Adjunctive Azithromycin in Non-Surgical Management of Chronic Periodontitis: A Multivariate Meta-Analysis.” *BMC Oral Health* 19, no. 1 (April 27, 2019): 65. <https://doi.org/10.1186/s12903-019-0754-0>.

Papageorgiou, S. N., P. N. Papageorgiou, J. Deschner, and W. Götz. “Comparative Effectiveness of Natural and Synthetic Bone Grafts in Oral and Maxillofacial Surgery Prior to Insertion of Dental Implants: Systematic Review and Network Meta-Analysis of Parallel and Cluster Randomized Controlled Trials.” *J Dent* 48 (May 2016): 1–8. <https://doi.org/10.1016/j.jdent.2016.03.010>.

Romandini, M., I. De Tullio, F. Congedi, Z. Kalemaj, M. D’Ambrosio, A. Laforí, C. Quaranta, J. Buti, and G. Perfetti. “Antibiotic Prophylaxis at Dental Implant Placement: Which Is the Best Protocol? A Systematic Review and Network Meta-Analysis.” *J Clin Periodontol* 46, no. 3 (March 2019): 382–95. <https://doi.org/10.1111/jcpe.13080>.

Tavelli, L., S. Barootchi, F. Cairo, G. Rasperini, K. Shedden, and H. L. Wang. “The Effect of Time on Root Coverage Outcomes: A Network Meta-Analysis.” *J Dent Res* 98, no. 11 (October 2019): 1195–1203. <https://doi.org/10.1177/0022034519867071>

**Primary reason for exclusion: not in English:**

Bai, Y., Y. L. Bai, J. Lai, and J. Huang. “[Efficacy of clarithromycin in the adjuvant treatment of chronic periodontitis: a Meta-analysis].” *Hua Xi Kou Qiang Yi Xue Za Zhi* 38, no. 3 (June 1, 2020): 290–96. <https://doi.org/10.7518/hxkq.2020.03.011>.

Bai, Y. L., T. J. Zheng, Z. W. Zhang, Y. Gan, and J. Huang. “[Efficacy of minocycline hydrochloride combined with flap surgery for chronic periodontitis: a Meta-analysis].” *Hua Xi Kou Qiang Yi Xue Za Zhi* 36, no. 4 (August 1, 2018): 421–27. <https://doi.org/10.7518/hxkq.2018.04.013>.

Hu, T., G. Ye, X. P. Fan, X. Y. Deng, W. H. Li, and X. R. Xiang. “[Platelet-rich plasma combined with demineralized freeze-dried bone allografts for periodontal regeneration in the treatment of periodontal defects: a meta-analysis].” *Shanghai Kou Qiang Yi Xue* 27, no. 5 (October 2018): 546–53.

Liu, Q. Y., Y. Zhang, J. Li, and X. R. Xiang. “[Locally delivered macrolides as an adjunct to non-surgical periodontal treatment of chronic periodontitis: a meta analysis].” *Shanghai Kou Qiang Yi Xue* 28, no. 2 (April 2019): 207–17.

Tan, J. P., Q. Jiang, M. F. Tan, P. Zhou, and F. J. Zhang. “[Efficacy of photodynamic therapy for peri-implantitis: A meta-analysis].” *Shanghai Kou Qiang Yi Xue* 28, no. 3 (June 2019): 330–326.

Xue, W., Z. Xiaobo, H. Bin, D. Yanhan, and L. Shumei. “[Decalcified freeze-dried bone allograft combined with rich platelet derivatives for the treatment of human periodontal intrabony defects: a Meta-analysis].” *Hua Xi Kou Qiang Yi Xue Za Zhi* 35, no. 6 (December 1, 2017): 636–42. <https://doi.org/10.7518/hxkq.2017.06.014>.

**Primary reason for exclusion: no SRs:**

Chambrone, L., R. C. N. de Castro Pinto, and L. A. Chambrone. “The Concepts of Evidence-Based Periodontal Plastic Surgery: Application of the Principles of Evidence-Based Dentistry for the Treatment of Recession-Type Defects.” *Periodontol 2000* 79, no. 1 (February 2019): 81–106. <https://doi.org/10.1111/prd.12248>.

Shiau, H. J. “Limited Evidence Suggests That Adjunctive Antimicrobial Photodynamic Therapy May Not Provide Additional Clinical Benefit to Conventional Instrumentation Strategy Alone in Periodontitis and Peri-Implantitis Patients.” *J Evid Based Dent Pract* 19, no. 4 (December 2019): 101346. <https://doi.org/10.1016/j.jebdp.2019.101346>.

**Primary reason for exclusion: no interventions:**

Chambrone, L., and D. N. Tatakis. “Long-Term Outcomes of Untreated Buccal Gingival Recessions: A Systematic Review and Meta-Analysis.” *J Periodontol* 87, no. 7 (July 2016): 796–808. <https://doi.org/10.1902/jop.2016.150625>.

Monje, A., K. T. Diaz, L. Aranda, A. Insua, A. Garcia-Nogales, and H. L. Wang. “Schneiderian Membrane Thickness and Clinical Implications for Sinus Augmentation: A Systematic Review and Meta-Regression Analyses.” *J Periodontol* 87, no. 8 (August 2016): 888–99. <https://doi.org/10.1902/jop.2016.160041>.

## List S5. Excluded SRs after assessment of the full text

**Primary reason for exclusion: data no displayed in forest plots:**

Cãlin, C., and I. Pãtraşcu. “Growth Factors and Beta-Tricalcium Phosphate in the Treatment of Periodontal Intraosseous Defects: A Systematic Review and Meta-Analysis of Randomised Controlled Trials.” *Arch Oral Biol* 66 (June 2016): 44–54. <https://doi.org/10.1016/j.archoralbio.2016.02.007>.

Coray, R., M. Zeltner, and M. Özcan. “Fracture Strength of Implant Abutments after Fatigue Testing: A Systematic Review and a Meta-Analysis.” *J Mech Behav Biomed Mater* 62 (September 2016): 333–46. <https://doi.org/10.1016/j.jmbbm.2016.05.011>.

de, N. Dias F. J., V. G. A. Pecorari, C. B. Martins, M. Del Fabbro, and M. Z. Casati. “Short Implants versus Bone Augmentation in Combination with Standard-Length Implants in Posterior Atrophic Partially Edentulous Mandibles: Systematic Review and Meta-Analysis with the Bayesian Approach.” *Int J Oral Maxillofac Surg* 48, no. 1 (January 2019): 90–96. <https://doi.org/10.1016/j.ijom.2018.05.009>.

Díaz-Faes, L., A. Fernández-Somoano, A. Magán-Fernández, and F. Mesa. “Efficacy of Regenerative Therapy in Aggressive Periodontitis: A Systematic Review and Meta-Analysis of Randomised Controlled Clinical Trials.” *Clin Oral Investig* 24, no. 4 (April 2020): 1369–78. <https://doi.org/10.1007/s00784-020-03237-0>.

Elangovan, S. “Dental Implants Placed in Alveolar Ridge Augmented Using Guided Bone Regeneration Procedure Performed Using Resorbable Collagen Membranes and Particulate Bone Grafts Using Simultaneous or Staged Approach Exhibit a High Survival Rate.” *J Evid Based Dent Pract* 18, no. 2 (June 2018): 173–75. <https://doi.org/10.1016/j.jebdp.2018.03.010>.

Elangovan, Satheesh. “Tunneling Technique in Conjunction With Autogenous Graft or Graft Substitutes Is a Predictable Surgical Approach to Achieve Root Coverage in Isolated or Multiple  Gingival Recession Defects.” *The Journal of Evidence-Based Dental Practice* 19, no. 2 (June 2019): 189–91. <https://doi.org/10.1016/j.jebdp.2019.05.012>.

Farano, V., J. C. Maurin, N. Attik, P. Jackson, B. Grosgogeat, and K. Gritsch. “Sol-Gel Bioglasses in Dental and Periodontal Regeneration: A Systematic Review.” *J Biomed Mater Res B Appl Biomater* 107, no. 4 (May 2019): 1210–27. <https://doi.org/10.1002/jbm.b.34214>.

Huang, J. P., J. M. Liu, Y. M. Wu, L. L. Chen, and P. H. Ding. “Efficacy of Xenogeneic Collagen Matrix in the Treatment of Gingival Recessions: A Systematic Review and Meta-Analysis.” *Oral Dis* 25, no. 4 (May 2019): 996–1008. <https://doi.org/10.1111/odi.12949>.

Jokstad, A., and J. Ganeles. “Systematic Review of Clinical and Patient-Reported Outcomes Following Oral Rehabilitation on Dental Implants with a Tapered Compared to a Non-Tapered Implant Design.” *Clin Oral Implants Res* 29 Suppl 16 (October 2018): 41–54. <https://doi.org/10.1111/clr.13128>.

Karl, M., and T. Albrektsson. “Clinical Performance of Dental Implants with a Moderately Rough (TiUnite) Surface: A Meta-Analysis of Prospective Clinical Studies.” *Int J Oral Maxillofac Implants* 32, no. 4 (August 2017): 717–34. <https://doi.org/10.11607/jomi.5699>.

Lee, C. T., Y. W. Chen, J. R. Starr, and S. K. Chuang. “Survival Analysis of Wide Dental Implant: Systematic Review and Meta-Analysis.” *Clin Oral Implants Res* 27, no. 10 (October 2016): 1251–64. <https://doi.org/10.1111/clr.12730>.

McGowan, K., T. McGowan, and S. Ivanovski. “Optimal Dose and Duration of Amoxicillin-plus-Metronidazole as an Adjunct to Non-Surgical Periodontal Therapy: A Systematic Review and Meta-Analysis of Randomized, Placebo-Controlled Trials.” *J Clin Periodontol* 45, no. 1 (January 2018): 56–67. <https://doi.org/10.1111/jcpe.12830>.

Monje, A., L. Aranda, K. T. Diaz, M. A. Alarcón, R. A. Bagramian, H. L. Wang, and A. Catena. “Impact of Maintenance Therapy for the Prevention of Peri-Implant Diseases: A Systematic Review and Meta-Analysis.” *J Dent Res* 95, no. 4 (April 2016): 372–79. <https://doi.org/10.1177/0022034515622432>.

Oh, T. J. “Adjunctive Use of Chlorhexidine Mouthwash to Nonsurgical Periodontal Therapy May Enhance Periodontal Pocket Depth Reduction.” *J Evid Based Dent Pract* 18, no. 4 (December 2018): 358–59. <https://doi.org/10.1016/j.jebdp.2018.10.004>.

Pommer, B., G. Mailath-Pokorny, R. Haas, D. Buseniechner, W. Millesi, and R. Fürhauser. “Extra-Short (< 7 Mm) and Extra-Narrow Diameter (< 3.5 Mm) Implants: A Meta-Analytic Literature Review.” *Eur J Oral Implantol* 11 Suppl 1 (2018): S137-s146.

Sanz-Sánchez, I., A. Ortiz-Vigón, I. Sanz-Martín, E. Figuero, and M. Sanz. “Effectiveness of Lateral Bone Augmentation on the Alveolar Crest Dimension: A Systematic Review and Meta-Analysis.” *J Dent Res* 94, no. 9 Suppl (September 2015): 128s–42. <https://doi.org/10.1177/0022034515594780>.

Sivaraman, K., A. Chopra, A. I. Narayan, and D. Balakrishnan. “Is Zirconia a Viable Alternative to Titanium for Oral Implant? A Critical Review.” *J Prosthodont Res* 62, no. 2 (April 2018): 121–33. <https://doi.org/10.1016/j.jpor.2017.07.003>.

Tatakis, D. N., and L. Chambrone. “The Effect of Suturing Protocols on Coronally Advanced Flap Root-Coverage Outcomes: A Meta-Analysis.” *J Periodontol* 87, no. 2 (February 2016): 148–55. <https://doi.org/10.1902/jop.2015.150394>.

Wang, J. C. “The Use of Amoxicillin (500 Mg) Plus Metronidazole (500 Mg) for 7 Days Adds Adjunctive Benefits for Nonsurgical Periodontal Therapy, but Limited Evidence Supports Higher/Longer Dose.” *J Evid Based Dent Pract* 18, no. 3 (September 2018): 249–51. <https://doi.org/10.1016/j.jebdp.2018.06.003>.

Wu, Y. C., L. K. Lin, C. J. Song, Y. X. Su, and Y. K. Tu. “Comparisons of Periodontal Regenerative Therapies: A Meta-Analysis on the Long-Term Efficacy.” *J Clin Periodontol* 44, no. 5 (May 2017): 511–19. <https://doi.org/10.1111/jcpe.12715>.

Zandbergen, D., D. E. Slot, R. Niederman, and F. A. Van der Weijden. “The Concomitant Administration of Systemic Amoxicillin and Metronidazole Compared to Scaling and Root Planing Alone in Treating Periodontitis: =a Systematic Review=.” *BMC Oral Health* 16 (February 29, 2016): 27. <https://doi.org/10.1186/s12903-015-0123-6>.

**Primary reason for exclusion: less then 4 studies included in meta-analysis:**

Cairo, F., L. Barbato, F. Selvaggi, M. G. Baielli, A. Piattelli, and L. Chambrone. “Surgical Procedures for Soft Tissue Augmentation at Implant Sites. A Systematic Review and Meta-Analysis of Randomized Controlled Trials.” *Clin Implant Dent Relat Res* 21, no. 6 (December 2019): 1262–70. <https://doi.org/10.1111/cid.12861>.

Chambrone, L., H. L. Wang, and G. E. Romanos. “Antimicrobial Photodynamic Therapy for the Treatment of Periodontitis and Peri-Implantitis: An American Academy of Periodontology Best Evidence Review.” *J Periodontol* 89, no. 7 (July 2018): 783–803. <https://doi.org/10.1902/jop.2017.170172>.

Cope, A. L., N. Francis, F. Wood, and I. G. Chestnutt. “Systemic Antibiotics for Symptomatic Apical Periodontitis and Acute Apical Abscess in Adults.” *Cochrane Database Syst Rev* 9, no. 9 (September 27, 2018): Cd010136. <https://doi.org/10.1002/14651858.CD010136.pub3>.

Fraga, R. S., L. A. A. Antunes, Kbfdc Fontes, E. C. Küchler, Nlpp Iorio, and L. S. Antunes. “Is Antimicrobial Photodynamic Therapy Effective for Microbial Load Reduction in Peri-Implantitis Treatment? A Systematic Review and Meta-Analysis.” *Photochem Photobiol* 94, no. 4 (July 2018): 752–59. <https://doi.org/10.1111/php.12901>.

Khouly, I., R. S. Braun, T. Silvestre, W. Musa, R. J. Miron, and A. Demyati. “Efficacy of Antibiotic Prophylaxis in Intraoral Bone Grafting Procedures: A Systematic Review and Meta-Analysis.” *Int J Oral Maxillofac Surg* 49, no. 2 (February 2020): 250–63. <https://doi.org/10.1016/j.ijom.2019.07.003>.

Novello, S., A. Debouche, M. Philippe, F. Naudet, and S. Jeanne. “Clinical Application of Mesenchymal Stem Cells in Periodontal Regeneration: A Systematic Review and Meta-Analysis.” *J Periodontal Res* 55, no. 1 (January 2020): 1–12. <https://doi.org/10.1111/jre.12684>.

Tampi, M. P., L. Pilcher, O. Urquhart, E. Kennedy, K. K. O’Brien, P. B. Lockhart, E. Abt, et al. “Antibiotics for the Urgent Management of Symptomatic Irreversible Pulpitis, Symptomatic Apical Periodontitis, and Localized Acute Apical Abscess: Systematic Review and Meta-Analysis-a Report of the American Dental Association.” *J Am Dent Assoc* 150, no. 12 (December 2019): e179–216. <https://doi.org/10.1016/j.adaj.2019.09.011>.

**Primary reason for exclusion: no access:**

Costa, Lfnp da, Cdsf Amaral, D. D. S. Barbirato, A. T. T. Leão, and M. F. Fogacci. “Chlorhexidine Mouthwash as an Adjunct to Mechanical Therapy in Chronic Periodontitis: A Meta-Analysis.” *J Am Dent Assoc* 148, no. 5 (May 2017): 308–18. <https://doi.org/10.1016/j.adaj.2017.01.021>.

Faggion, C. M. “Laser Therapy as an Adjunct Treatment for Peri-Implant Mucositis and Peri-Implantitis Provides No Extra Benefit for Most Clinical Outcomes.” *J Evid Based Dent Pract* 19, no. 2 (June 2019): 203–6. <https://doi.org/10.1016/j.jebdp.2019.05.008>.

Liu, Y., B. Hu, J. Zhou, W. Li, Q. Liu, and J. Song. “The Effect of Enamel Matrix Derivative Alone Versus in Combination with Alloplastic Materials to Treat Intrabony Defects: A Meta-Analysis.” *Int J Periodontics Restorative Dent* 37, no. 4 (August 2017): e224–33. <https://doi.org/10.11607/prd.2900>.

Mokcheh, A., H. Jegham, and S. Turki. “Short Implants as an Alternative to Sinus Lift for the Rehabilitation of Posterior Maxillary Atrophies: Systematic Review and Meta-Analysis.” *J Stomatol Oral Maxillofac Surg* 120, no. 1 (February 2019): 28–37. <https://doi.org/10.1016/j.jormas.2018.11.006>.

Xu, C., Q. Wang, J. Chen, Y. Wu, and L. Zhao. “Collagen Matrix for Periodontal Plastic Surgery Procedures: A Meta-Analysis Update.” *Int J Periodontics Restorative Dent* 39, no. 4 (August 2019): e129–55. <https://doi.org/10.11607/prd.3814>.

**Primary reason for exclusion: data only displayed visually:**

Keestra, J. A., I. Grosjean, W. Coucke, M. Quirynen, and W. Teughels. “Non-Surgical Periodontal Therapy with Systemic Antibiotics in Patients with Untreated Aggressive Periodontitis: A Systematic Review and Meta-Analysis.” *J Periodontal Res* 50, no. 6 (December 2015): 689–706. <https://doi.org/10.1111/jre.12252>.

Matarasso, M., V. Iorio-Siciliano, A. Blasi, L. Ramaglia, G. E. Salvi, and A. Sculean. “Enamel Matrix Derivative and Bone Grafts for Periodontal Regeneration of Intrabony Defects. A Systematic Review and Meta-Analysis.” *Clin Oral Investig* 19, no. 7 (September 2015): 1581–93. <https://doi.org/10.1007/s00784-015-1491-7>.

Wessing, B., S. Lettner, and W. Zechner. “Guided Bone Regeneration with Collagen Membranes and Particulate Graft Materials: A Systematic Review and Meta-Analysis.” *Int J Oral Maxillofac Implants* 33, no. 1 (February 2018): 87–100. <https://doi.org/10.11607/jomi.5461>.

**Primary reason for exclusion: only evaluation of risk factors:**

Antonoglou, G. N., A. Stavropoulos, M. D. Samara, A. Ioannidis, G. I. Benic, S. N. Papageorgiou, and G. K. Sándor. “Clinical Performance of Dental Implants Following Sinus Floor Augmentation: A Systematic Review and Meta-Analysis of Clinical Trials with at Least 3 Years of Follow-Up.” *Int J Oral Maxillofac Implants* 33, no. 3 (June 2018): e45–65. <https://doi.org/10.11607/jomi.6417>.

**Primary reason for exclusion: bad scan quality/ unreadable:**

Panda, S., J. Doraiswamy, S. Malaiappan, S. S. Varghese, and M. Del Fabbro. “Additive Effect of Autologous Platelet Concentrates in Treatment of Intrabony Defects: A Systematic Review and Meta-Analysis.” *J Investig Clin Dent* 7, no. 1 (February 2016): 13–26. <https://doi.org/10.1111/jicd.12117>.
